# Supplementary material for: Cytokine concentrations in people with eating disorders: A comprehensive updated systematic review and meta-analysis
Source: Commun Med (Lond). 2025 Oct 1;5:408. doi: 10.1038/s43856-025-01122-z (PMC12488998; doi:10.1038/s43856-025-01122-z)
Supplement: Supplementary file 2 — Supplementary Information [file 43856_2025_1122_MOESM2_ESM.pdf]

Supplementary Materials for "Cytokine concentrations in people with eating disorders: A comprehensive updated systematic review and meta-analysis"

Johanna L. Keeler [1*]

Charlotte Bovenberg [2]

Hubertus Himmerich [1,3]

Janet Treasure [1,3]

Ben Carter [4]

Ulrike Schmidt [1,3]

Bethan Dalton [3,5]

Affiliations

[1] Centre for Research in Eating and Weight Disorders, Department of Psychological Medicine, Institute of Psychiatry, Psychology and Neuroscience, King's College London, London, UK.

[2] Department of Psychosis Studies, Institute of Psychiatry, Psychology and Neuroscience, King's College London, London, UK.

[3] South London and Maudsley NHS Foundation Trust, Bethlem Royal Hospital, Monks Orchard Road, Beckenham, Kent, UK.

[4] Department of Biostatistics and Health Informatics, Institute of Psychiatry, Psychology and Neuroscience, King's College London, London, UK.

[5] Department of Psychology, Institute of Psychiatry, Psychology and Neuroscience, King's College London, London, UK.

*Correspondence to: Dr Johanna Keeler, johanna.keeler@kcl.ac.uk; Centre for Research in Eating and Weight Disorders, Department of Psychological Medicine, Institute of Psychiatry, Psychology and Neuroscience, King's College London, London, UK, SE5 8AF.

Quality Assessment: Newcastle-Ottawa Scale adapted for cross-sectional studies

Studies are allocated stars (up to a total of 11) based on meeting certain criteria, described below. The greater number of stars allocated indicates a higher quality.

Selection: (Maximum 5 stars)

1) Representativeness of the sample:

   a) Truly representative of the average in the target population (all subjects or random sampling) **

   b) Somewhat representative of the average in the target population (non-random sampling) *

   c) Selected group of users or no description of the sampling strategy.

2) Sample size:

   a) Justified and satisfactory *

   b) Not justified

3) Ascertainment of the exposure (disease i.e. ED):

   a) Validated measurement tool in both ED and HC group **

   b) Non-validated measurement tool, but the tool is available or described in both ED and HC group or validated measurement tool described in one group (ED or HC) *

   c) No description of the measurement tool or non-validated measurement tool, but the tool is available or described for only one group (ED or HC)

Comparability: (Maximum 3 stars)

1) The subjects in different outcome groups are comparable, based on the study design. Confounding factors are controlled through pre-analysis techniques such as sample matching, data cleaning or eligibility criteria.

   a) The study controls for the most important factor (age) *

   b) The study controls for an additional important factor (sex) *

   c) The study controls for an additional important factor (e.g., smoking status, presence of inflammatory condition, use of medication) *

Outcome: (Maximum 3 stars)

1) Measurement of outcome:

a) Validated measurement method (interassay CV included) **

b) Non-validated measurement method, but the method is available or described *

c) No description of the measurement tool

2) Statistical test:

a) The statistical test used to analyse the data is clearly described and appropriate, and the measurement of the association is presented, including confidence intervals and the probability level (p value). *

b) The statistical test is not appropriate, not described or incomplete.

This scale was adapted from the Newcastle-Ottawa Quality Assessment Scale for cohort studies to perform a quality assessment of cross-sectional studies for use in the following systematic review: Herzog R, Álvarez-Pasquin MJ, Díaz C, Del Barrio JL, Estrada JM, Gil Á, 2013. Are healthcare workers' intentions to vaccinate related to their knowledge, beliefs and attitudes? A systematic review. BMC Public Health. 13, 154. We further adapted the scale from ascertainment of outcome (e.g. independent blind assessment/record linkage/self-report) to measurement of outcome to account for the methods used in this field of research.

<u>Summary of quality assessment</u>

About half of the studies ensured representativeness of their sample (n=21), and few used a validated measurement tool in both the ED and HC groups to ascertain diagnosis (n=16). Some studies either used a non-validated measurement tool or only used the tool in the ED group (n=12). Fifteen studies had no description of the measurement tool (n=14). Three studies provided justification for their sample size (n=3). In terms of comparability, most studies accounted for age as a confounding variable (n=26), and almost all studies accounted for an additional confounding factor (e.g. BMI, sex; smoking status; n=41). Few studies explicitly reported using a validated method to measure the inflammatory factor level, including the interassay CV (n=19), 23 studies used a non-validated measurement method that was described and one study did not describe their measurement tool. All studies presented the measurement of the association with CIs and the probability level (*p*-values) (n=43).

Narrative summary of additional studies not included in meta-analysis

Anorexia nervosa versus healthy controls. In people with AN compared to controls, concentrations of IL-21 and IL-23 were found to be increased, and concentrations of Eotaxin, Eotaxin-3 and IL-1α were increased in some studies but were similar to controls in other studies. Cytokines found to be decreased in AN included IL-9, IL-12B, IL-17F, IL-23, and monocyte chemotactic protein (MCP)-3. There were mixed evidence for decreases in AN in some studies and no difference in comparison to controls in other studies, in concentrations of IL-2, IL-12/IL-23p40, IL-16, IL-17A, IL-18, IL-31, MIP-1ß and TNF-ß. Additionally, the following cytokines were found to not differ between AN and controls: granulocyte-macrophage colony-stimulating factor (GM-CSF), IL-1, IL-5, IL-12p70, IL-13, IL-17C, IL-22, IL-27, IL-33, IP-10, MCP-2, MCP-4, macrophage-derived chemokine (MDC), MIP-1α, MIP-3α, and thymus and activation-regulation chemokine (TARC).

Anorexia nervosa baseline to follow-up. Longitudinally, concentrations of 23 cytokines were found not to change, although notably these findings were largely from a study whereby full weight gain did not occur longitudinally[1]. These cytokines included: Eotaxin, Eotaxin-3, GM-CSF, IL-1α, IL-2, IL-3, IL-4, IL-5, IL-8, IL-10, IL-12/IL-23p40, IL-12p70; IL-13; IL-15; IL-16; IL-17A, IP-10, MCP-1, MCP-4, MIP-1ß, MIP-1α, TARC and TNF-ß. This same study found a longitudinal increase in IL-7 after a 24-week follow-up. For interferon (IFN)-γ, there were mixed results whereby one study found no longitudinal change, and another study found an increase after 10 weeks of nutritional reinstatement including yogurt intake, but a decrease after 10 weeks of nutritional reinstatement including milk intake[2].

Individuals recovered from anorexia nervosa versus healthy controls. People recovered from AN (recAN) showed increased concentrations of IL-12p70 and decreased concentrations of Eotaxin-3, IL-17A, MDC and TNF-α in comparison with healthy controls. Moreover, for IL-7, MCP-1, MIP-1α and MIP-1ß, some studies found decreased concentrations in recAN and some found no difference between recAN and controls. Concentrations of 23 other cytokines were similar between recAN and controls, which included: Eotaxin, GM-CSF, IFN-γ, IL-2, IL-4, IL-8, IL-10, IL-12/IL-23p40, IL-12B, IL-13, IL-17C, IL-18, IL-22, IL-27, IL-31, IP-10, MCP-2, MCP-3, MCP-4, MIP-3α, TARC, transforming growth factor (TGF)-ß, and TNF-ß.

Bulimia nervosa versus healthy controls. Concentrations of IL-10 and IL-13 were found to be decreased in people with BN compared to controls. Concentrations of IL-4, IL-7, TGF-ß and IL-1ß were found to be similar between BN and controls, although one study found an increase in IL-1ß in people with BN.

Binge eating disorder versus healthy controls. In BED, it was found that concentrations of IL-10 were decreased in comparison to controls, and concentrations of TNF-α were increased. Concentrations of IL-1α, IL-1ß, IL-2, IL-4, IL-6 and IL-8 were comparable between BED and controls. However, all findings were based on one study only.

Otherwise-specified feeding and eating disorder versus healthy controls. One study measured IL-6 concentrations in women with OSFED, which was numerically higher than controls (SMD = 0.52), although inferential statistics for this comparison were not presented[3].

Figure S1. Funnel plot for cross-sectional meta-analysis of tumour necrosis factor-α (TNF-α) concentrations between anorexia nervosa and healthy controls.

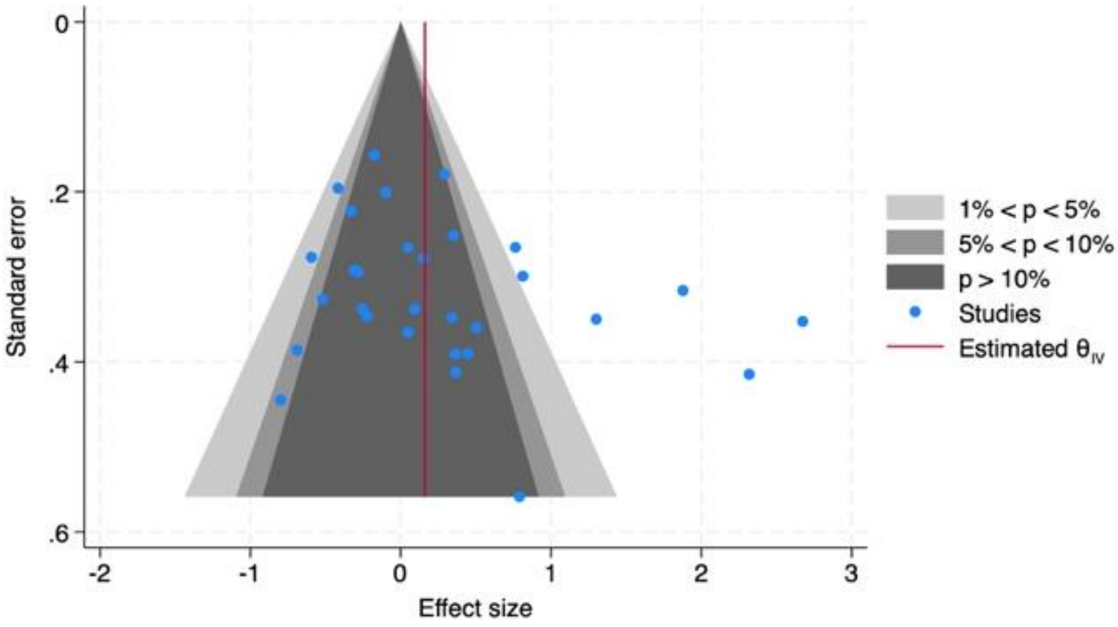

Abbreviations: *p* = level of statistical significance of the individual study.

Figure S2. Funnel plot for cross-sectional meta-analysis of interleukin-6 (IL-6) concentrations between anorexia nervosa and healthy controls

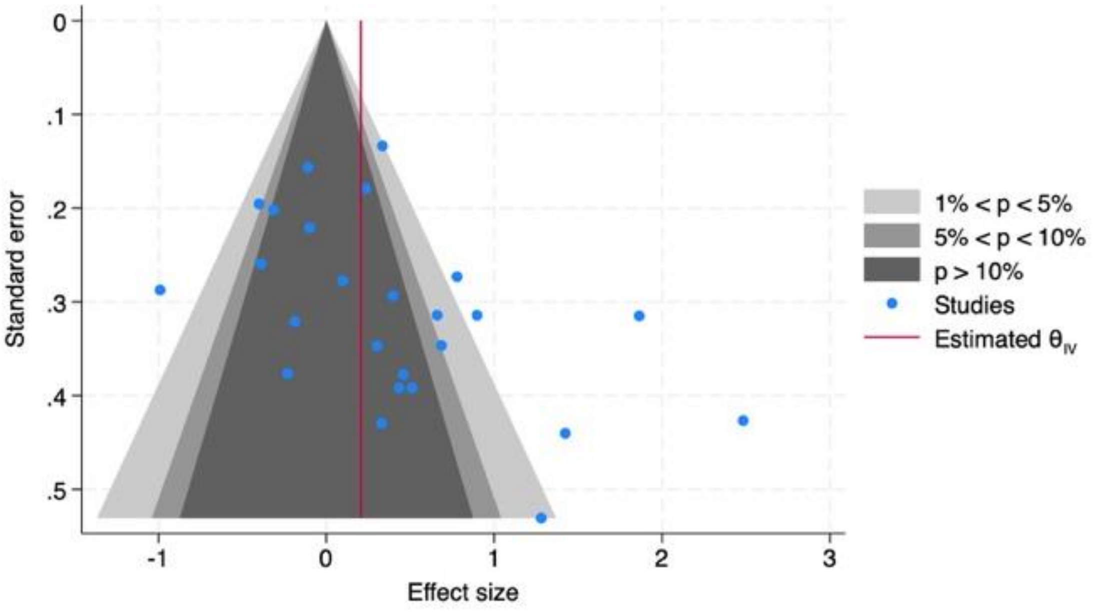

Abbreviations: *p* = level of statistical significance of the individual study.

Figure S3. Funnel plot for cross-sectional meta-analysis of interleukin-1β (IL-1β) concentrations between anorexia nervosa and healthy controls

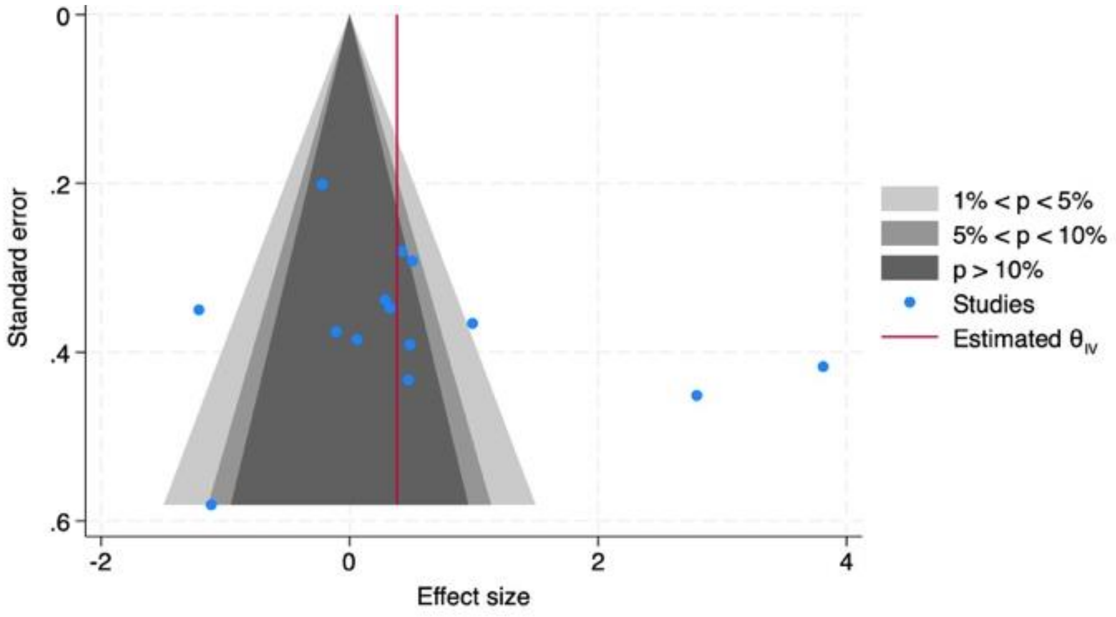

Abbreviations: *p* = level of statistical significance of the individual study.

Figure S4. Funnel plot for cross-sectional meta-analysis of interleukin-10 (IL-10) concentrations between anorexia nervosa and healthy controls

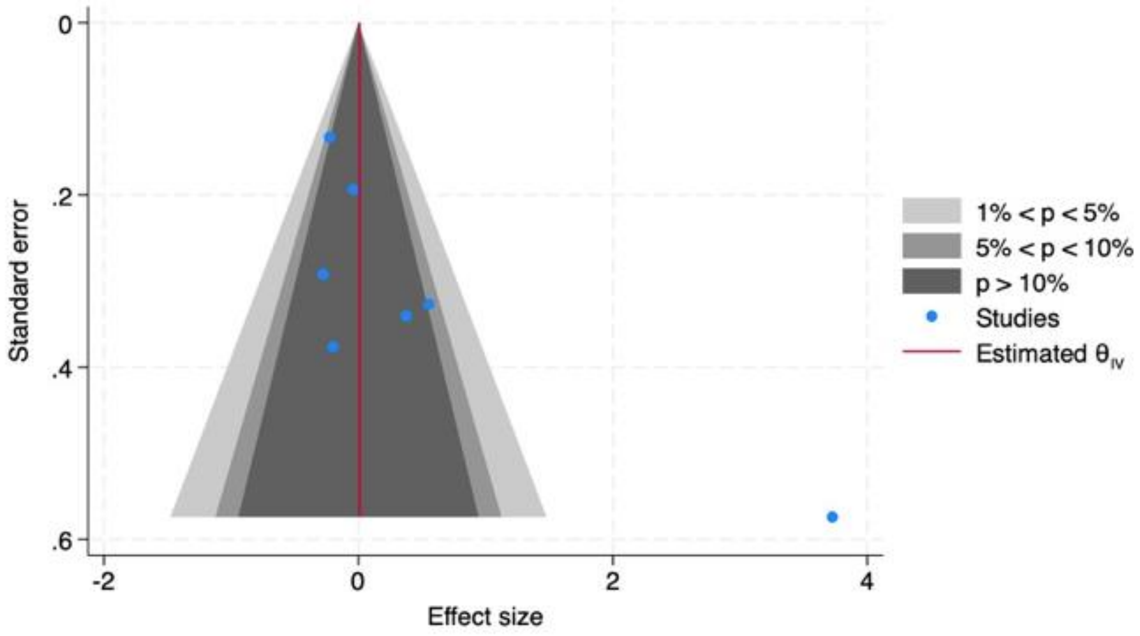

Abbreviations: *p* = level of statistical significance of the individual study.

Figure S5. Funnel plot for cross-sectional meta-analysis of interferon-γ (IFN-γ) concentrations between anorexia nervosa and healthy controls.

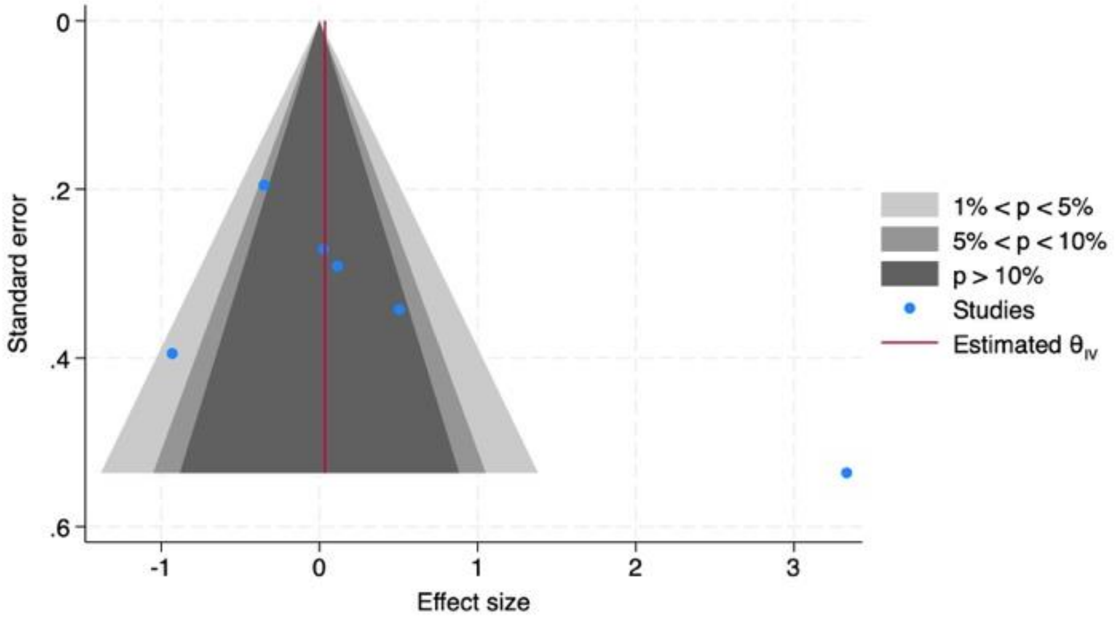

Abbreviations: *p* = level of statistical significance of the individual study.

Figure S6. Funnel plot for cross-sectional meta-analysis of interleukin-8 (IL-8) concentrations between anorexia nervosa and healthy controls

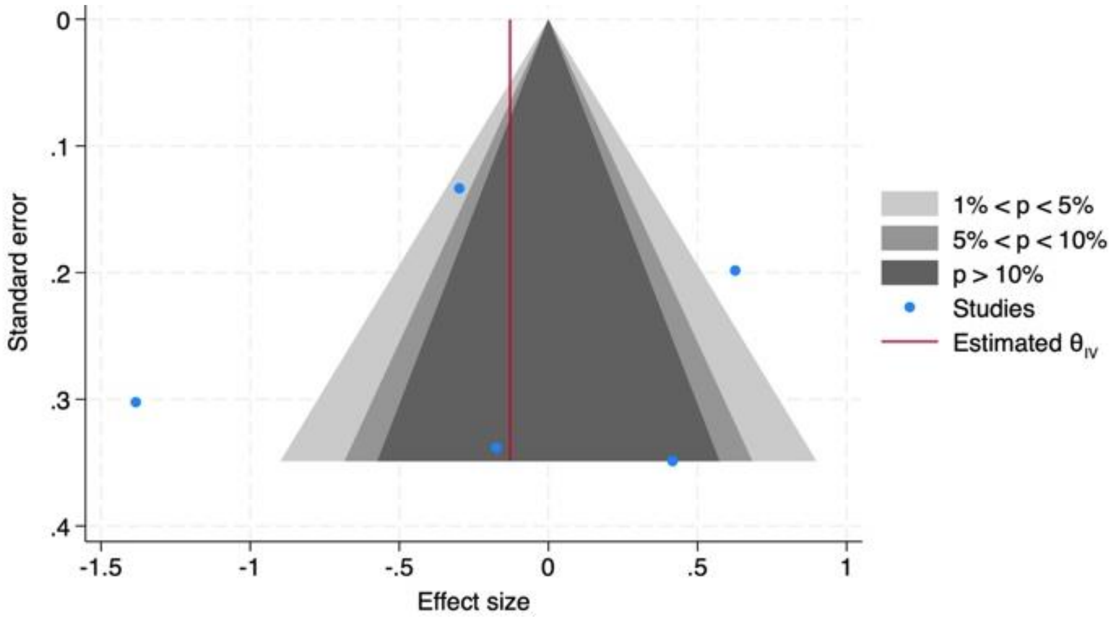

Abbreviations: *p* = level of statistical significance of the individual study.

Figure S7. Funnel plot for cross-sectional meta-analysis of monocyte chemoattractant protein-1 (MCP-1) concentrations between anorexia nervosa and healthy controls

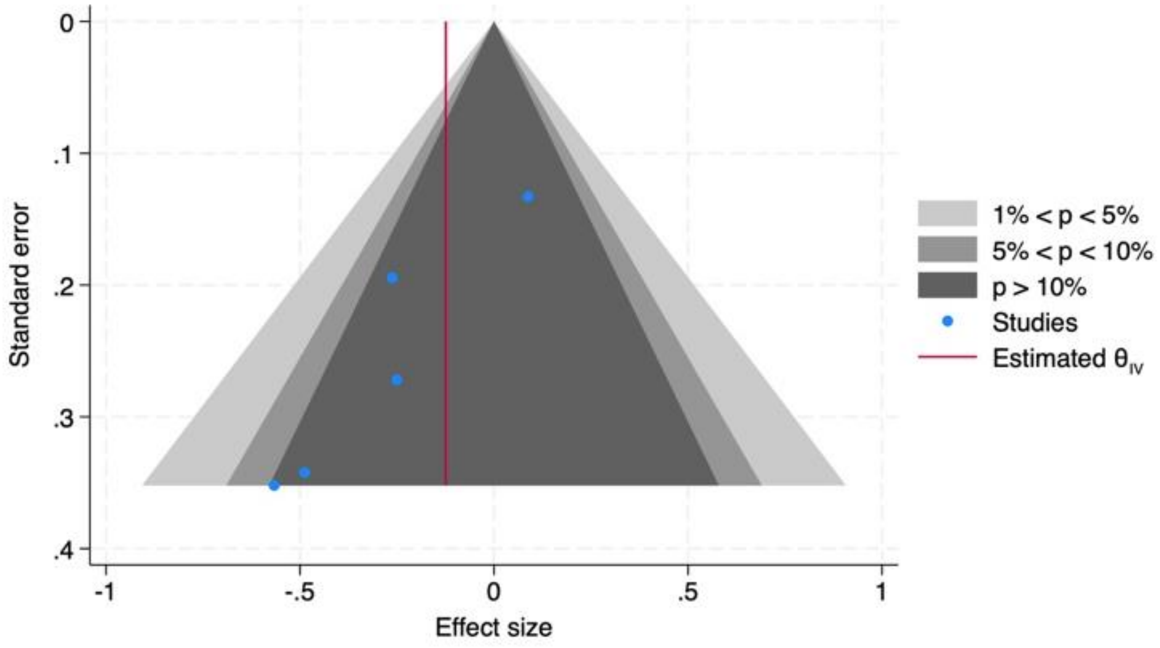

Abbreviations: *p* = level of statistical significance of the individual study.

Figure S8. Funnel plot for cross-sectional meta-analysis of transforming growth factor-β (TGF-β) concentrations between anorexia nervosa and healthy controls

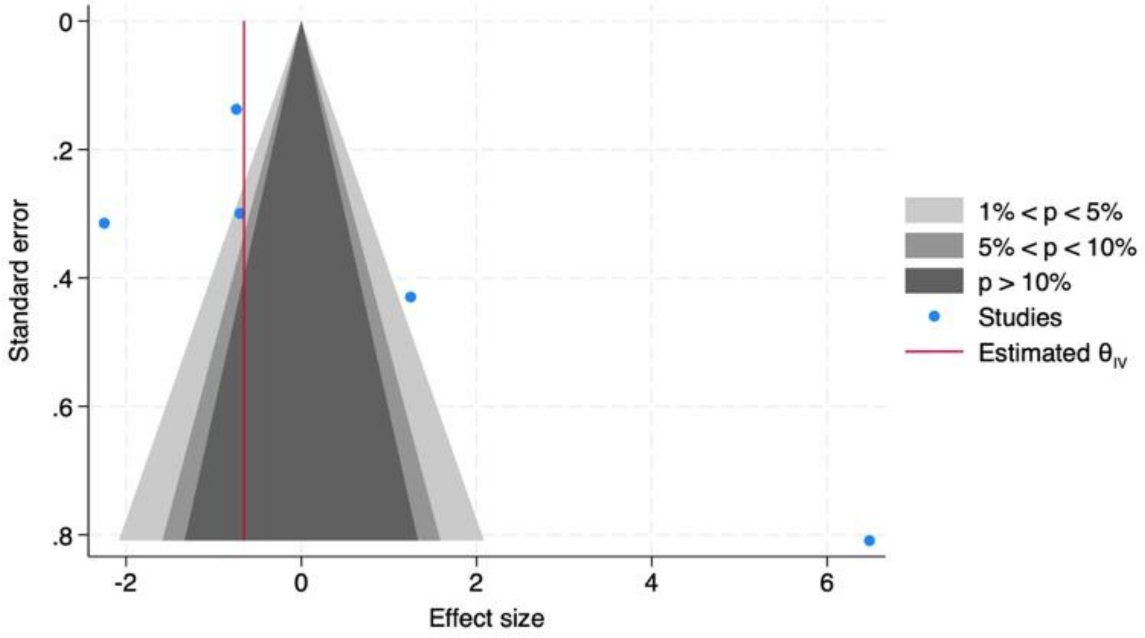

Abbreviations: *p* = level of statistical significance of the individual study.

Figure S9. Funnel plot for cross-sectional meta-analysis of interleukin-4 (IL-4) concentrations between anorexia nervosa and healthy controls

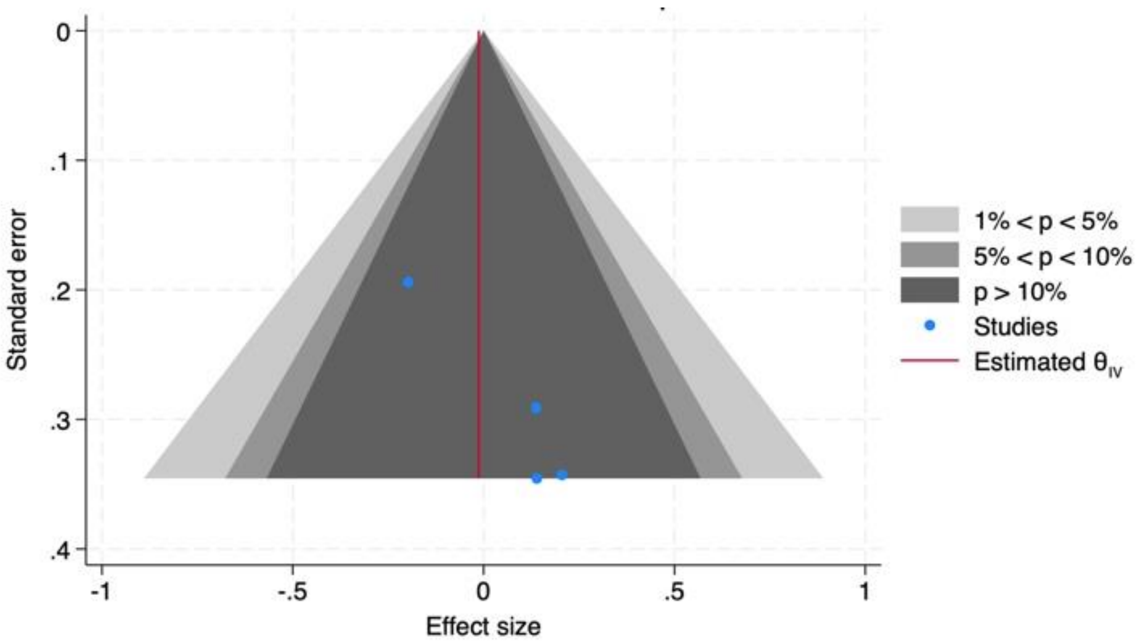

Abbreviations: *p* = level of statistical significance of the individual study.

Figure S10. Funnel plot for cross-sectional meta-analysis of interleukin-7 (IL-7) concentrations between anorexia nervosa and healthy controls

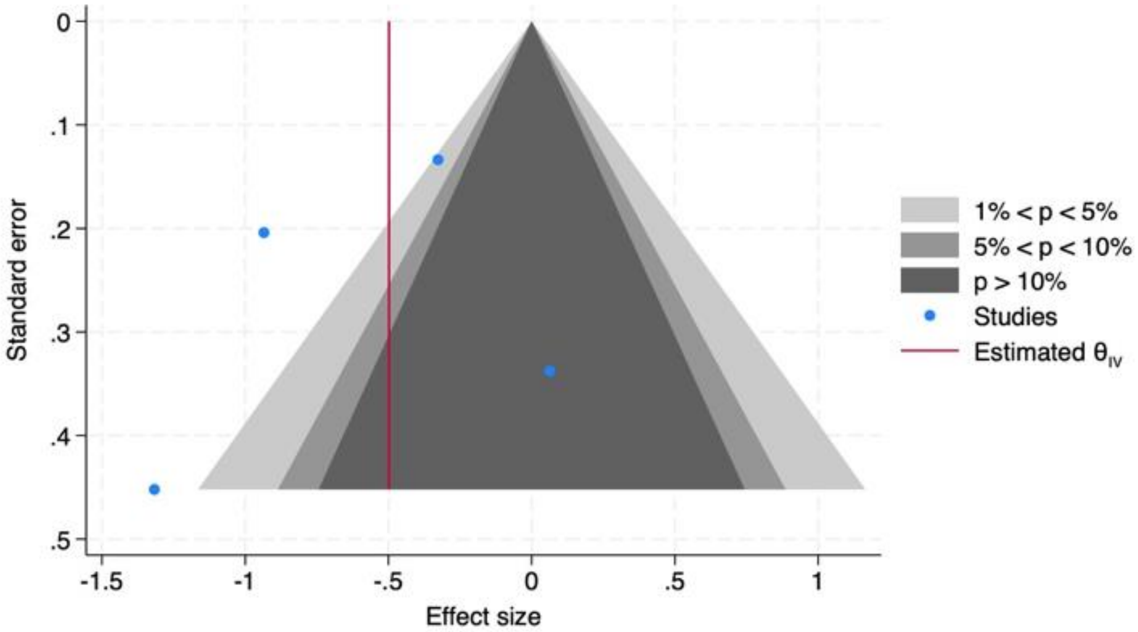

Abbreviations: *p* = level of statistical significance of the individual study.

Figure S11. Funnel plot for cross-sectional meta-analysis of interleukin-15 (IL-15) concentrations between anorexia nervosa and healthy controls

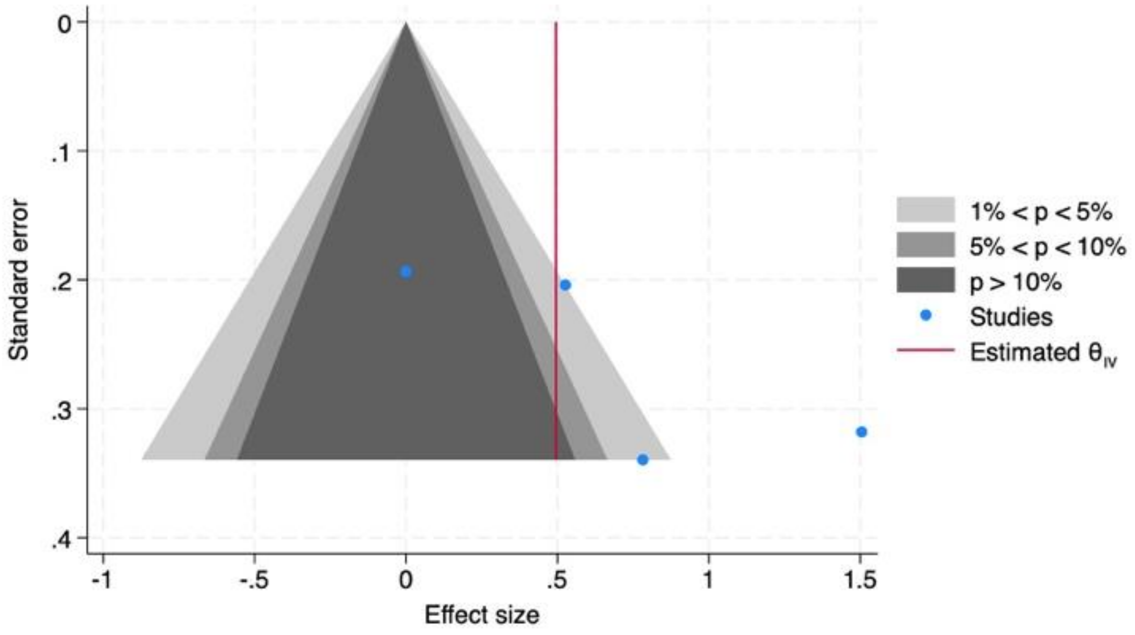

Abbreviations: *p* = level of statistical significance of the individual study.

Figure S12. Funnel plot for cross-sectional meta-analysis of interleukin-6 (IL-6) concentrations between bulimia nervosa and healthy controls

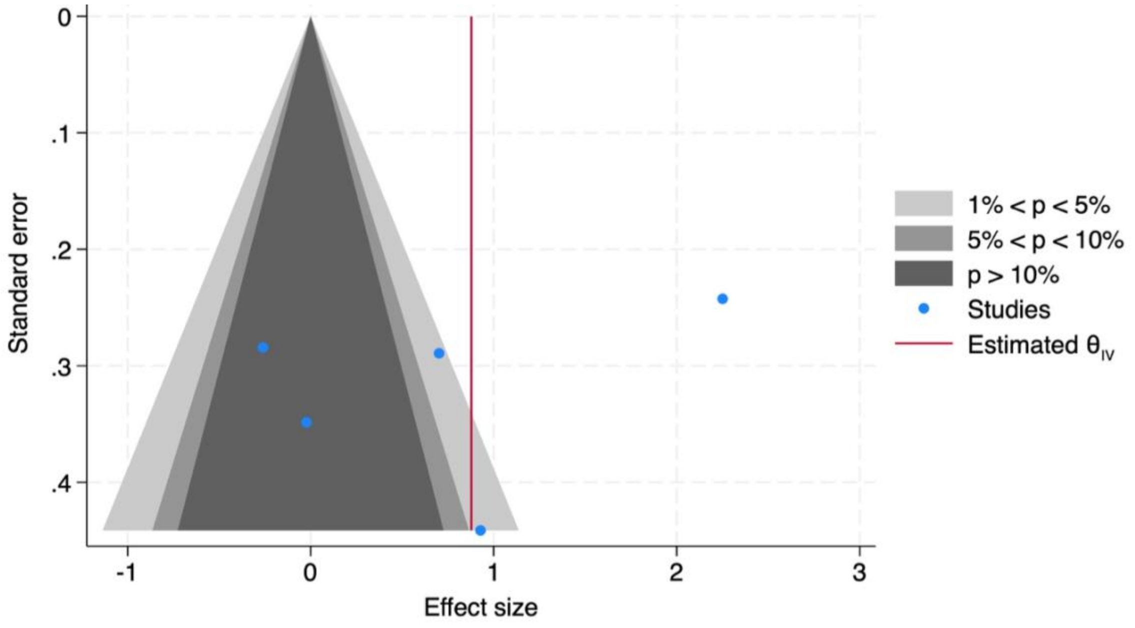

Abbreviations: *p* = level of statistical significance of the individual study.

Figure S13. Funnel plot for cross-sectional meta-analysis of tumour necrosis factor-α (TNF-α) concentrations between bulimia nervosa and healthy controls

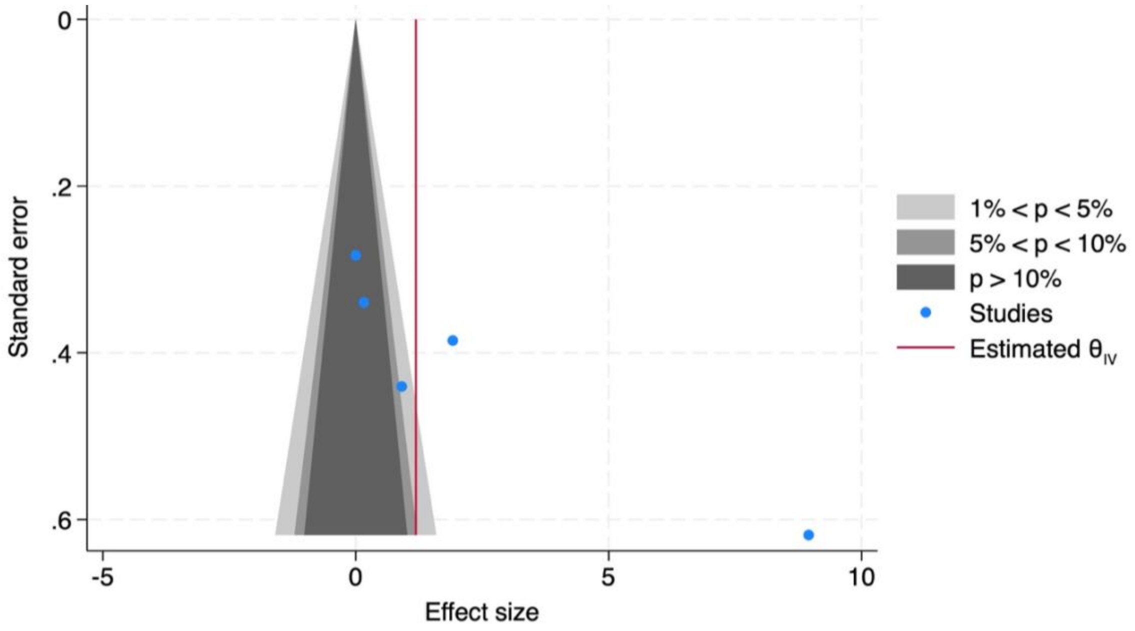

Abbreviations: *p* = level of statistical significance of the individual study.

Figure S14. Funnel plot for meta-analysis of tumour necrosis factor-α (TNF-α) concentrations between anorexia nervosa at baseline and follow-up

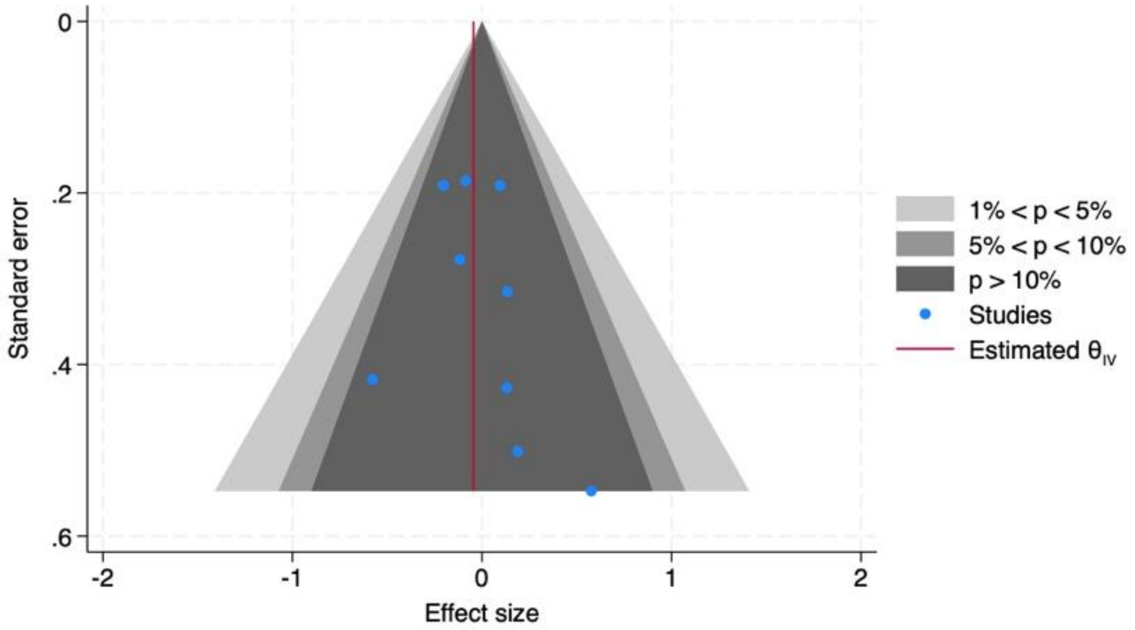

Abbreviations: *p* = level of statistical significance of the individual study.

Figure S15. Funnel plot for meta-analysis of interleukin-1β (IL-β) concentrations between anorexia nervosa at baseline and follow-up

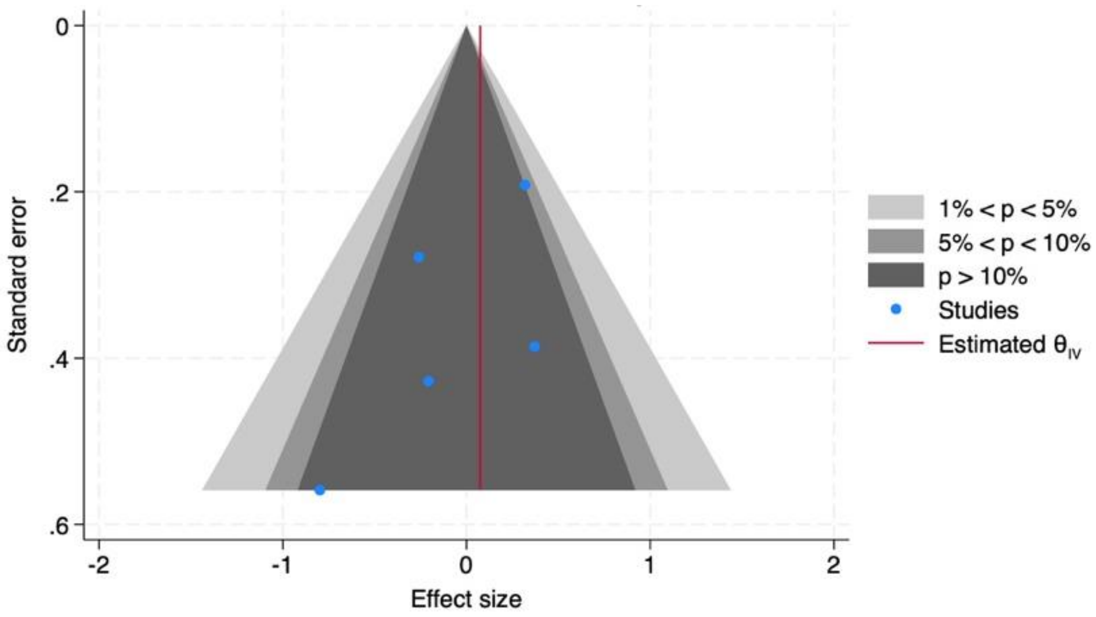

Abbreviations: *p* = level of statistical significance of the individual study.

Figure S16. Funnel plot for meta-analysis of interleukin-6 (IL-6) concentrations between anorexia nervosa at baseline and follow-up.

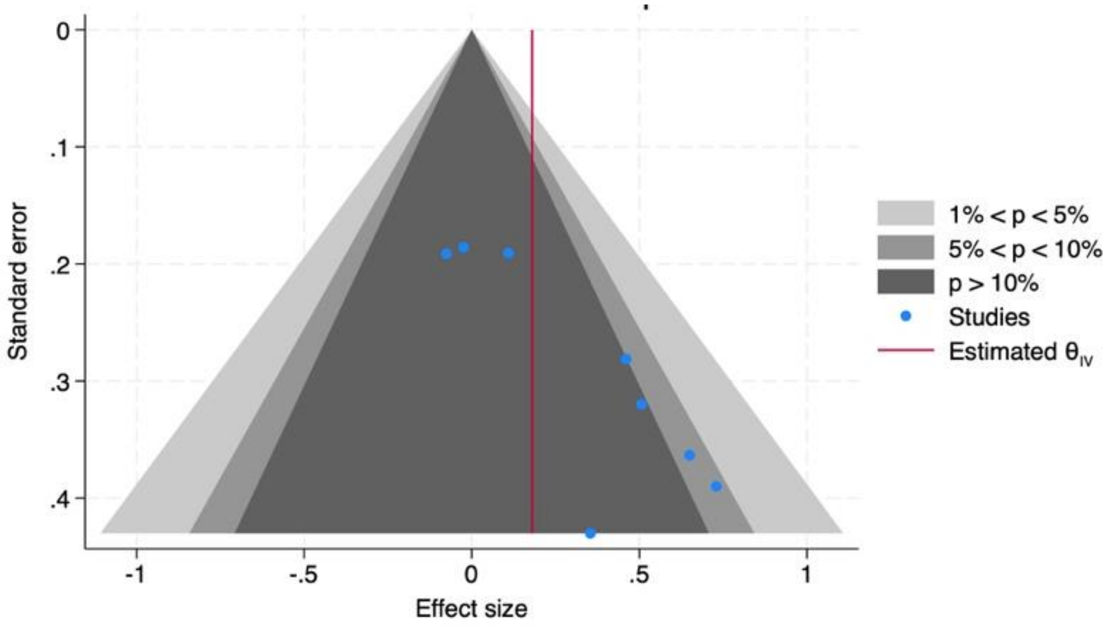

Abbreviations: *p* = level of statistical significance of the individual study.

Figure S17. Cumulative forest plot for meta-analysis of tumour necrosis factor-α (TNF-α) concentrations between anorexia nervosa and healthy controls, descending according to Newcastle-Ottawa (NOS) quality assessment score.

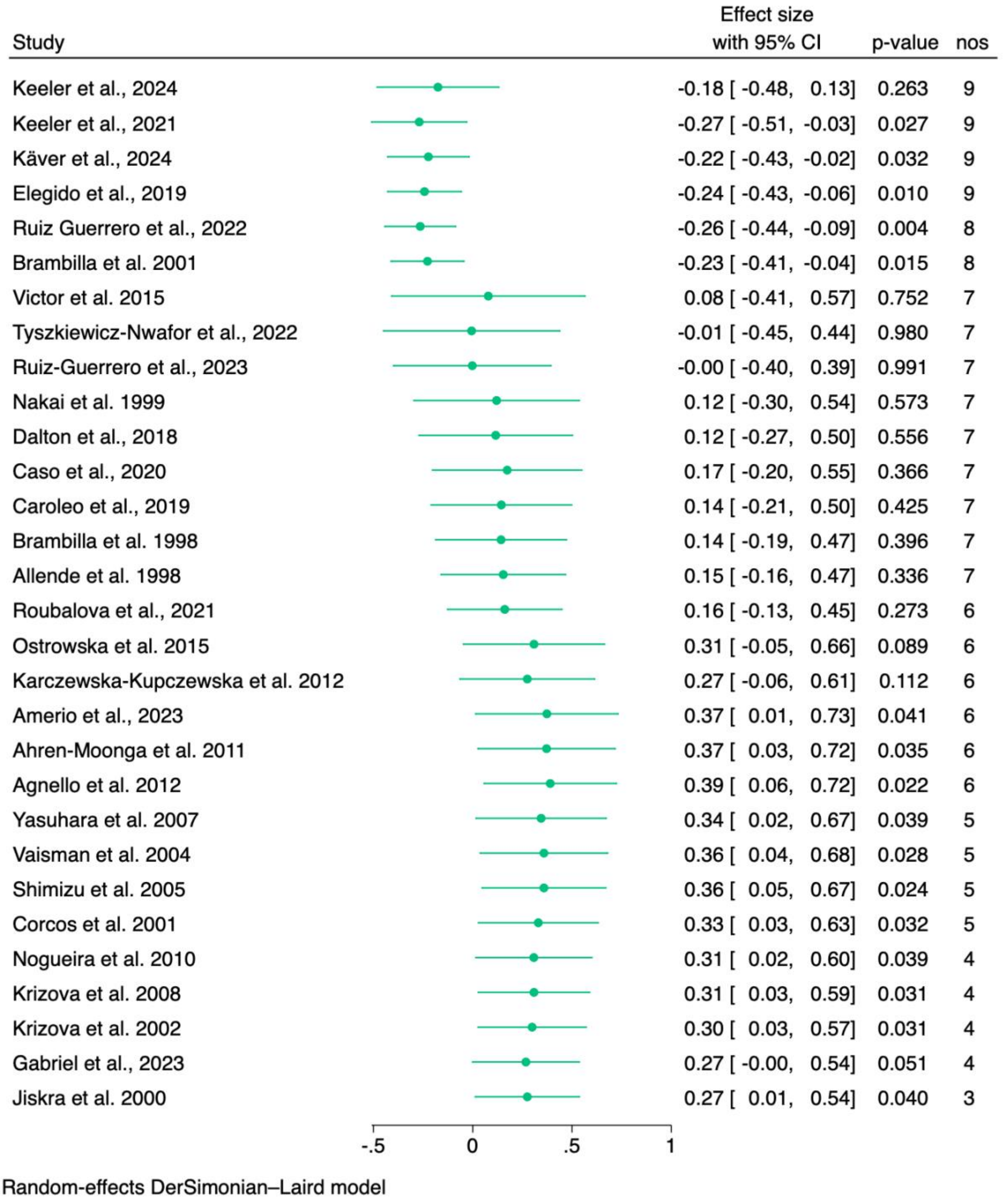

Abbreviations: CI = confidence intervals. Error bars reflect 95% CIs.

Figure S18. Cumulative forest plot for meta-analysis of tumour necrosis factor-α (TNF-α) concentrations between anorexia nervosa and healthy controls, descending according to year of publication.

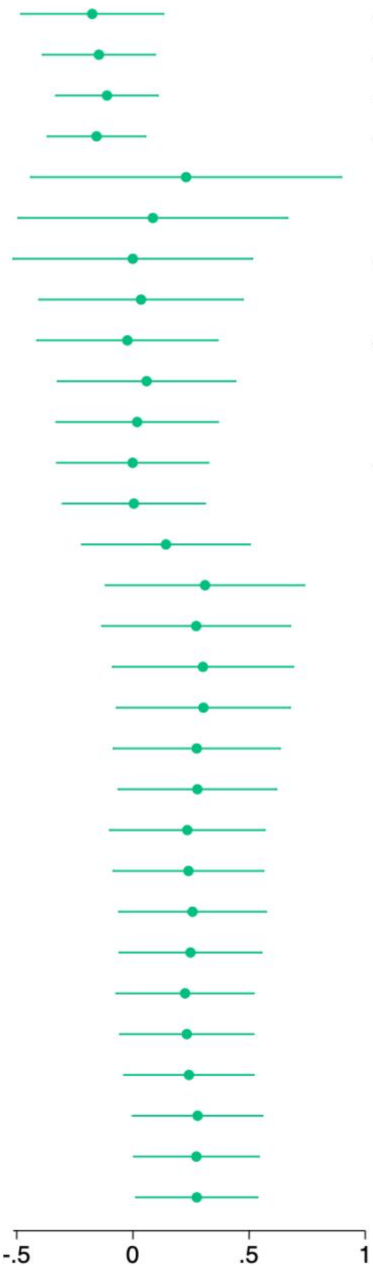

Study	Effect size with 95% CI	p-value	year
Keeler et al., 2024	-0.18 [-0.48, 0.13]	0.263	2024
Käver et al., 2024	-0.15 [-0.39, 0.10]	0.236	2024
Ruiz-Guerrero et al., 2023	-0.11 [-0.33, 0.11]	0.319	2023
Gabriel et al., 2023	-0.16 [-0.37, 0.05]	0.146	2023
Amerio et al., 2023	0.23 [-0.44, 0.90]	0.502	2023
Tyszkiewicz-Nwafor et al., 2022	0.09 [-0.49, 0.67]	0.772	2022
Ruiz Guerrero et al., 2022	-0.00 [-0.52, 0.51]	0.998	2022
Roubalova et al., 2021	0.03 [-0.40, 0.47]	0.876	2021
Keeler et al., 2021	-0.02 [-0.41, 0.37]	0.905	2021
Caso et al., 2020	0.06 [-0.32, 0.44]	0.763	2020
Elegido et al., 2019	0.02 [-0.33, 0.37]	0.918	2019
Caroleo et al., 2019	-0.00 [-0.33, 0.33]	0.995	2019
Dalton et al., 2018	0.00 [-0.30, 0.31]	0.979	2018
Victor et al. 2015	0.14 [-0.22, 0.50]	0.441	2015
Ostrowska et al. 2015	0.31 [-0.12, 0.74]	0.155	2015
Karczewska-Kupczewska et al. 2012	0.27 [-0.13, 0.68]	0.188	2012
Agnello et al. 2012	0.30 [-0.09, 0.69]	0.129	2012
Ahren-Moonga et al. 2011	0.30 [-0.07, 0.68]	0.111	2011
Nogueira et al. 2010	0.27 [-0.08, 0.63]	0.133	2010
Krizova et al. 2008	0.28 [-0.06, 0.62]	0.109	2008
Yasuhara et al. 2007	0.23 [-0.10, 0.57]	0.169	2007
Shimizu et al. 2005	0.24 [-0.08, 0.56]	0.147	2005
Vaisman et al. 2004	0.26 [-0.06, 0.57]	0.112	2004
Krizova et al. 2002	0.25 [-0.06, 0.55]	0.112	2002
Corcos et al. 2001	0.22 [-0.07, 0.52]	0.137	2001
Brambilla et al. 2001	0.23 [-0.06, 0.52]	0.114	2001
Jiskra et al. 2000	0.24 [-0.04, 0.52]	0.091	2000
Nakai et al. 1999	0.28 [-0.00, 0.56]	0.051	1999
Brambilla et al. 1998	0.27 [0.00, 0.54]	0.047	1998
Allende et al. 1998	0.27 [0.01, 0.54]	0.040	1998

Random-effects DerSimonian–Laird model

Abbreviations: CI = confidence intervals. Error bars reflect 95% CIs.

Figure S19. Cumulative forest plot for meta-analysis of interleukin-6 (IL-6) concentrations between anorexia nervosa and healthy controls, descending according to Newcastle-Ottawa (NOS) quality assessment score.

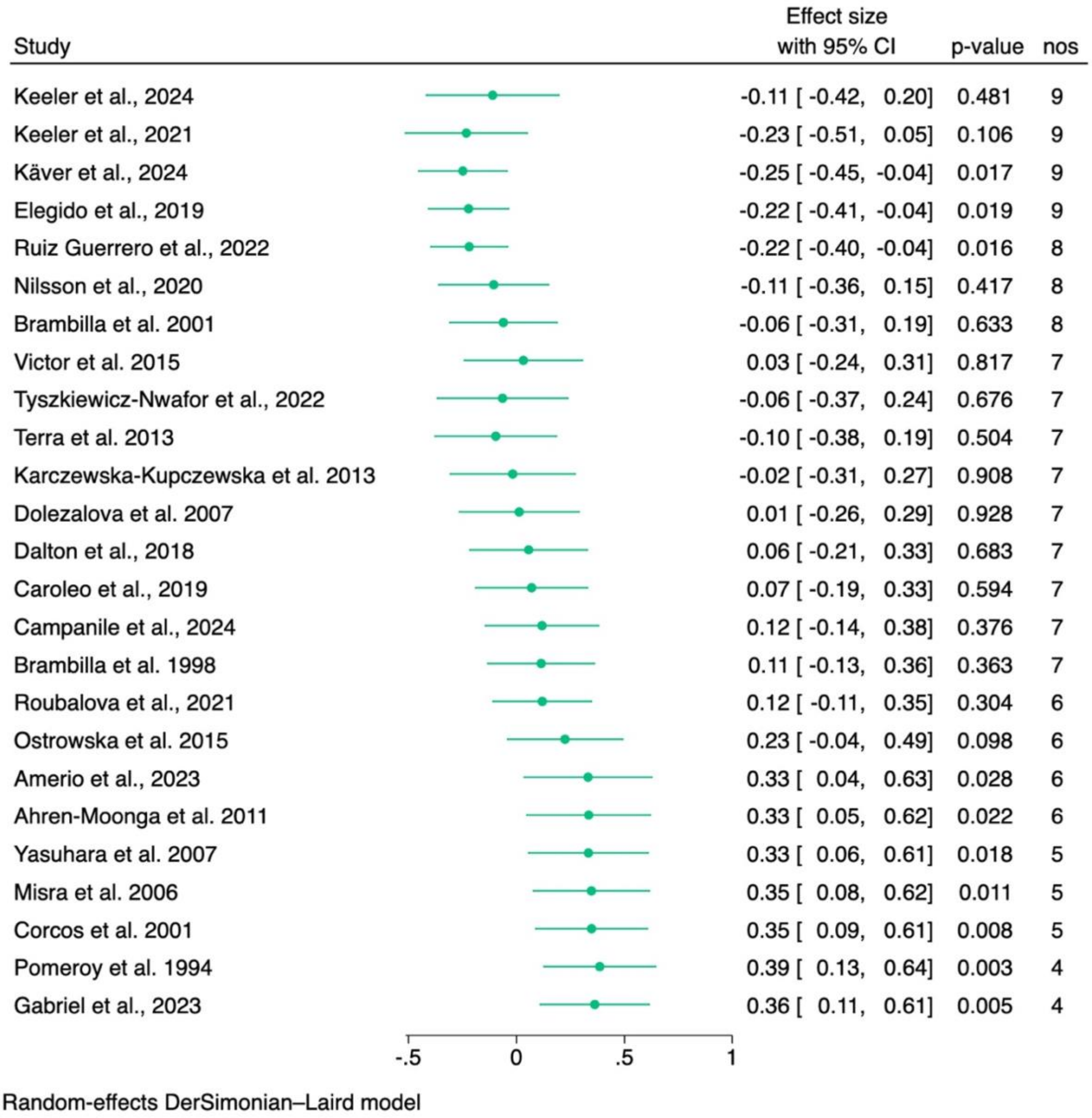

Abbreviations: CI = confidence intervals. Error bars reflect 95% CIs.

Figure S20. Cumulative forest plot for meta-analysis of interleukin-6 (IL-6) concentrations between anorexia nervosa and healthy controls, descending according to year of publication.

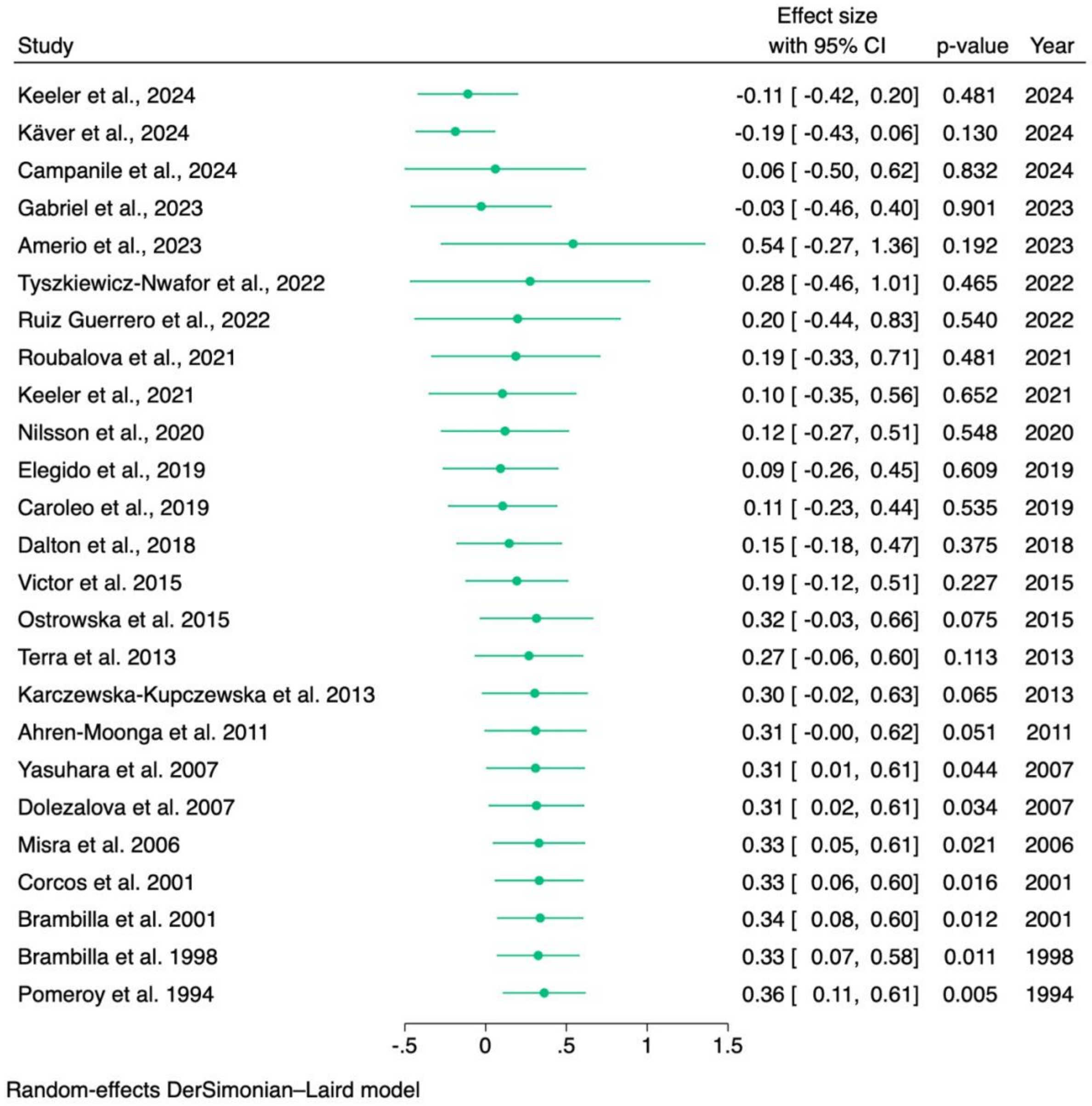

Abbreviations: CI = confidence intervals. Error bars reflect 95% CIs.

Figure S21. Forest plot of standardized mean difference in tumour necrosis factor-α (TNF-α) between anorexia nervosa (AN) participants and healthy controls stratified by AN subtype. Zero is the line of no effect, and points to the right of zero indicate an elevation of the cytokine in AN compared to HCs.

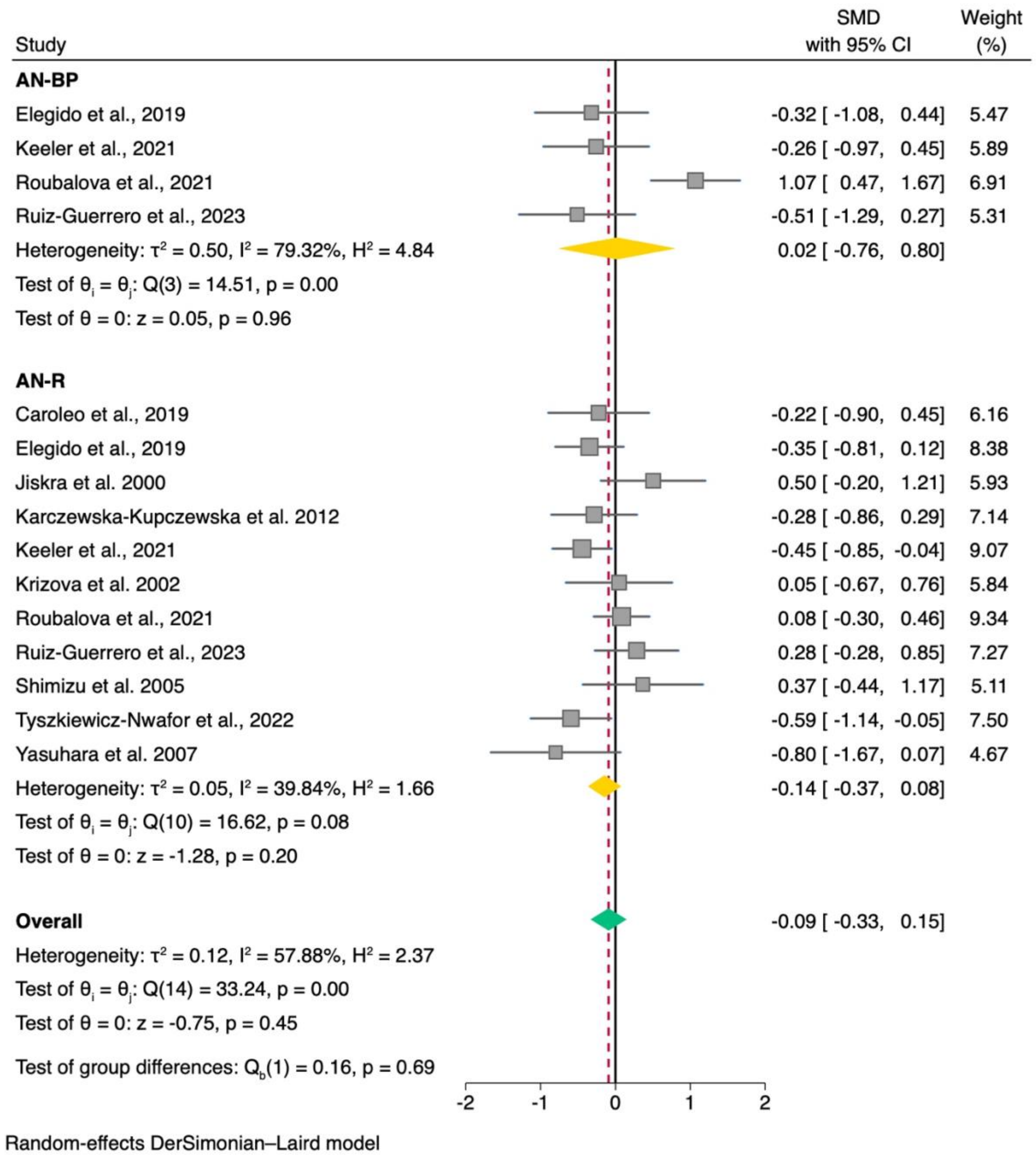

Abbreviations: AN-BP = AN binge-purge subtype; AN-R = AN restricting subtype; CI = confidence intervals; SMD = standardized mean difference. Error bars reflect 95% CIs.

Metaforest moderator analysis for meta-analysis of TNF-α concentrations between anorexia nervosa and healthy controls

To further investigate sources of heterogeneity, a random-effects MetaForest analysis was conducted after removing study outliers. Irrelevant moderators were identified using 100-fold replicated feature selection, with only those showing positive variable importance in more than 50% of replications carried forward. Eight moderators were retained for model tuning (replicated variable importance metrics shown in Figure S22). The main analysis consisted of 10,000 regression trees with random-effect weights, four candidate variables per split and a minimum of five cases per terminal node. The final model showed positive estimates of explained variance in new data (R_{oob}^2 = 0.22; R_{cv}^2 = 0.83). The model identified the following moderators as most important in explaining the effect size: age of the AN sample, age of the HC sample, percentage of the sample using psychotropic medication, age group, percentage of the sample who were smokers, sample type (serum/plasma), study quality score and year of publication. The relative variable importance of these moderators is shown in Figure S23. The marginal relationship of each moderator with the effect size, averaging over all values of other moderators, is illustrated in Figure S24. These moderators were entered into meta-regressions but none were significantly associated (Table S1).

Figure S22. Replicated MetaForest for variable preselection, meta-analysis of tumour necrosis factor (TNF)-α concentrations between anorexia nervosa and healthy controls after removing study outliers.

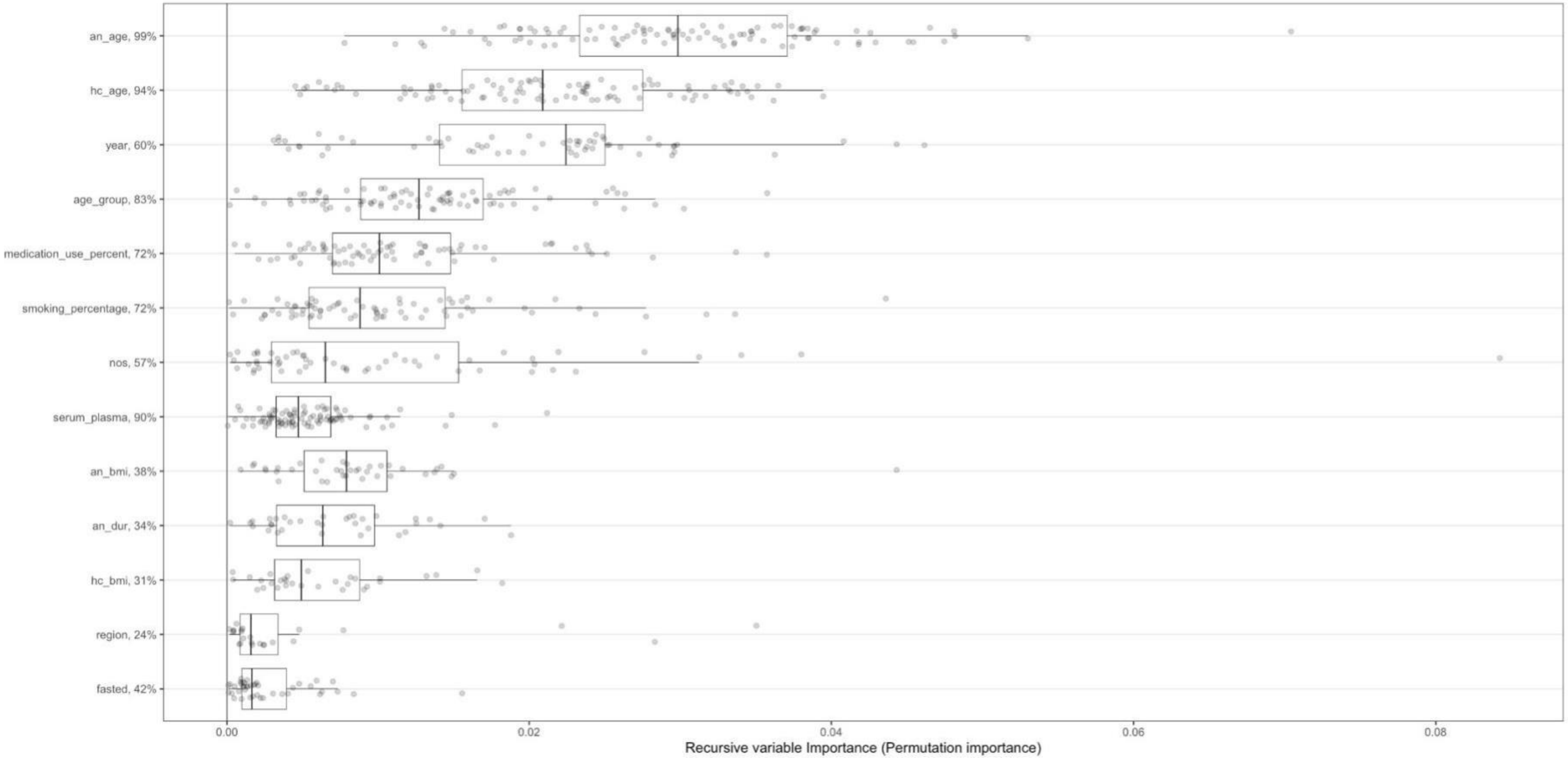

Abbreviations: AN = anorexia nervosa; BMI = body mass index; dur = illness duration; HC = healthy controls; nos = Newcastle-Ottowa Score. Error bars reflect 95% confidence intervals.

Figure S23. Variable importance for final model, meta-analysis of tumour necrosis factor (TNF)-α concentrations between anorexia nervosa and healthy controls after removing study outliers.

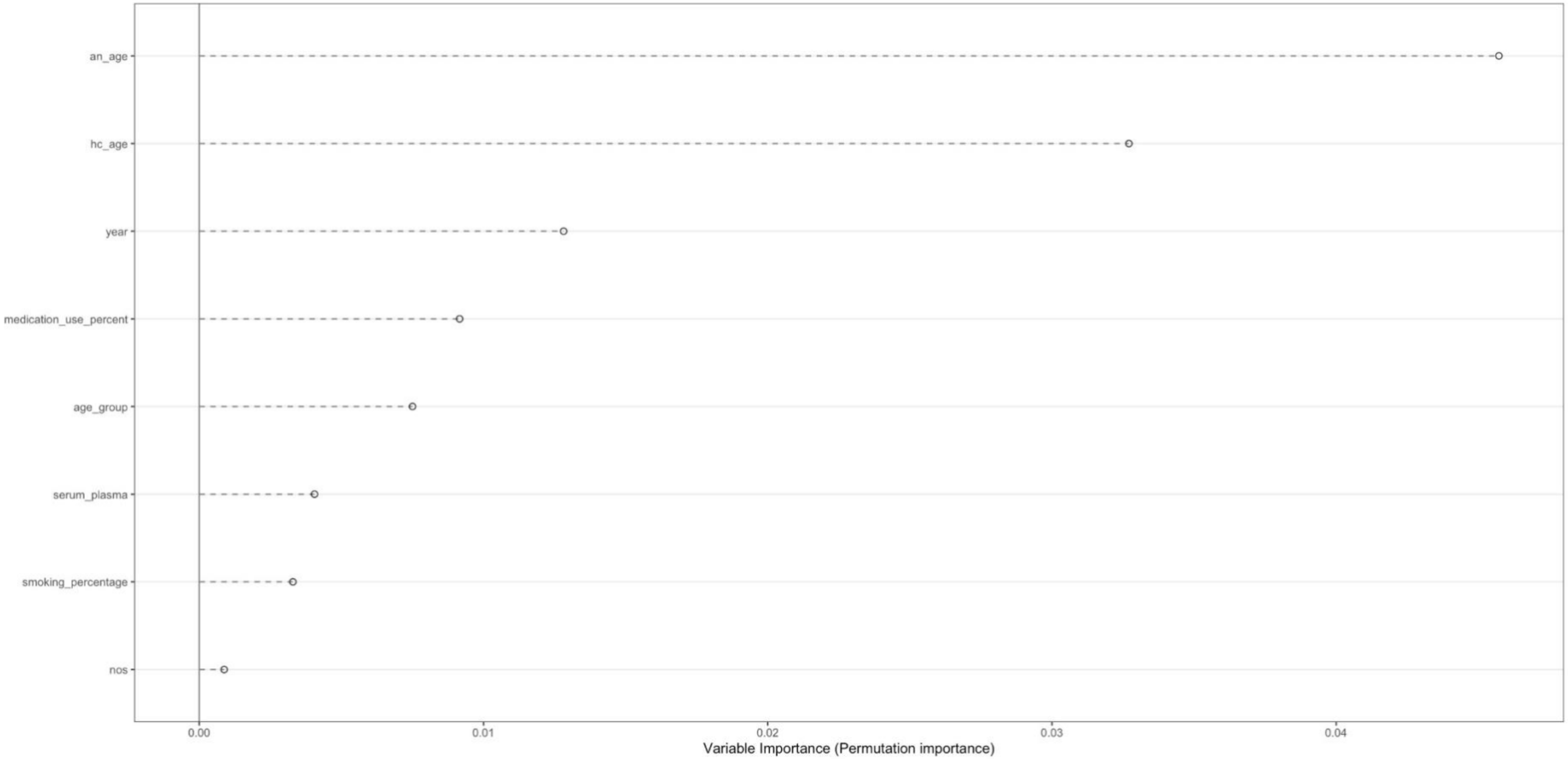

Abbreviations: AN = anorexia nervosa; HC = healthy controls; nos = Newcastle-Ottowa Score.

Figure S24. Partial dependence plots, meta-analysis of tumour necrosis factor (TNF)-α concentrations between anorexia nervosa and healthy controls after removing study outliers.

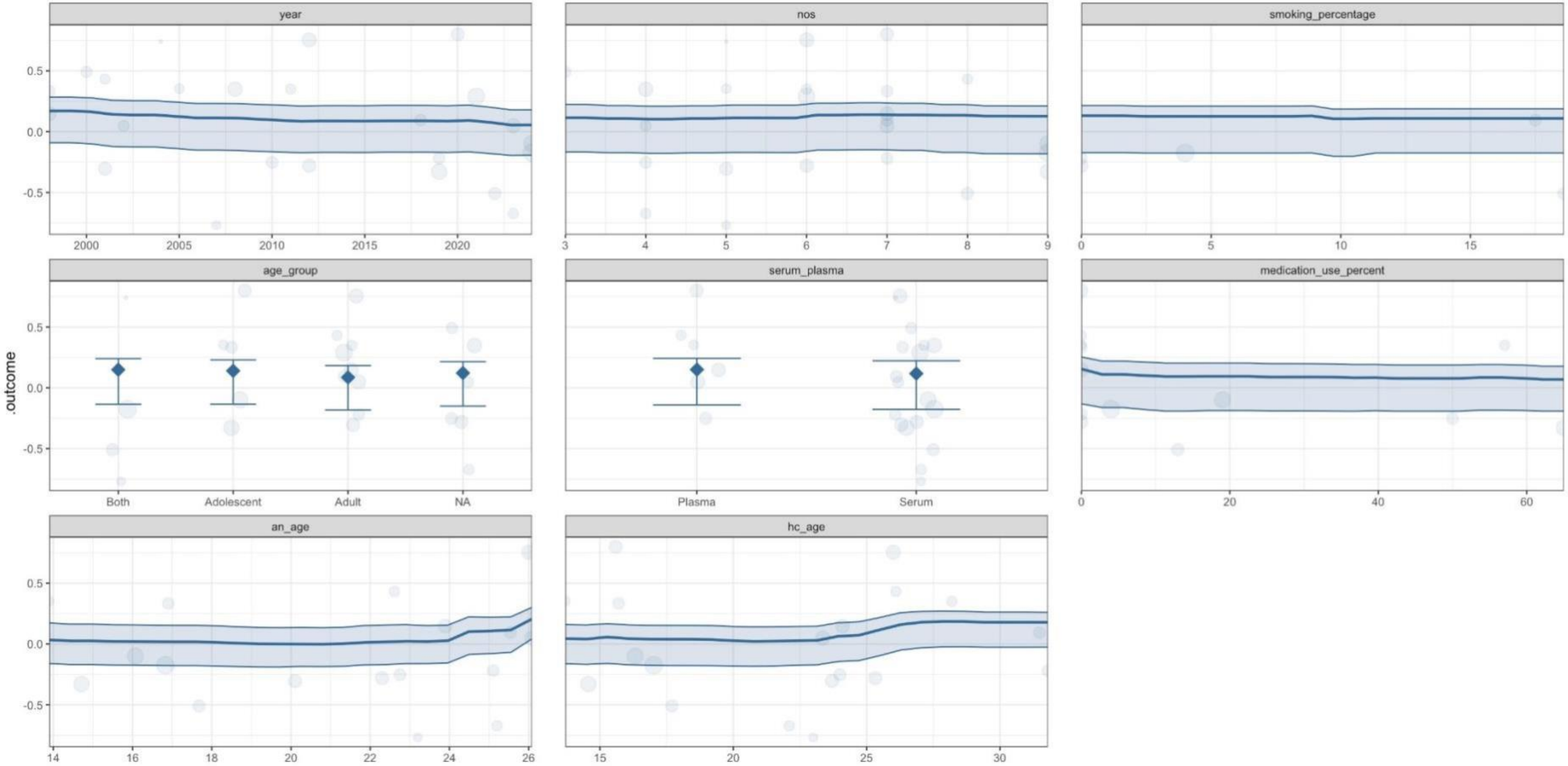

Abbreviations: AN = anorexia nervosa; HC = healthy controls; NA = missing; nos = Newcastle-Ottowa Score. Error bars reflect 95% confidence intervals for the model's predictions as each value of the predictor variable.

Table S1. Results of exploratory individual meta-regressions for moderators selected from MetaForest analysis on standardised mean differences in tumour necrosis factor (TNF)-α concentrations between anorexia nervosa and healthy controls, after removing six study outliers [4-9].

Variable	Estimate	SE	z-value	p-value	(95% CI)
Study year	-0.01	0.01	-1.40	0.162	-0.03, 0.01
Study NOS score	-0.04	0.05	-0.85	0.395	-0.13, 0.05
Percentage of sample who smoke	-0.001	0.02	-0.05	0.964	-0.04, 0.03
Age group*Adults[a]	0.05	0.21	0.23	0.818	-0.37, 0.47
Age group*Both[a]	-0.39	0.27	-1.45	0.147	-0.91, 0.14
Percentage of sample using psychotropic medication	-0.01	0.004	-1.28	0.200	-0.01, 0.003
Sample type*Serum[b]	-0.24	0.19	-1.28	0.201	-0.62, 0.13
Mean age (AN)	0.02	0.02	0.93	0.355	-0.02, 0.06
Mean age (HC)	0.003	0.02	0.15	0.880	-0.03, 0.04

[a]Reference category was adolescents. [b]Reference category was plasma. Abbreviations: AN = anorexia nervosa; BMI = body mass index; CI = confidence intervals; HC = healthy controls; NOS = Newcastle-Ottawa Scale; SE = standard error.

Figure S25. Forest plot of standardized mean difference in interleukin-6 (IL-6) between anorexia nervosa (AN) participants and healthy controls stratified by AN subtype. Zero is the line of no effect, and points to the right of zero indicate an elevation of the cytokine in AN compared to HCs.

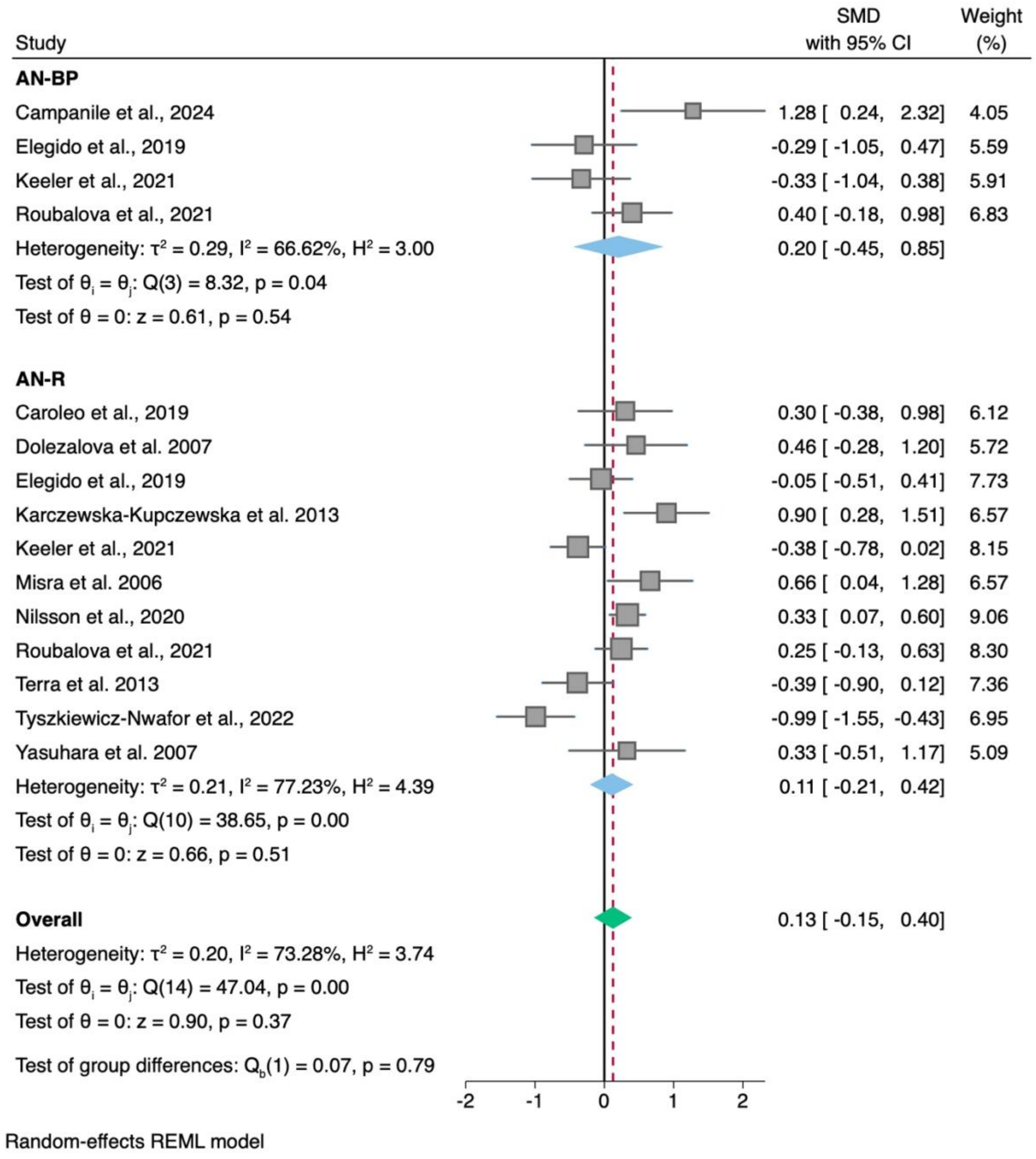

Abbreviations: AN-BP = AN binge-purge subtype; AN-R = AN restricting subtype; CI = confidence intervals; SMD = standardized mean difference. Error bars reflect 95% CIs.

MetaForest moderator analysis for meta-analysis of IL-6 concentrations between anorexia nervosa and healthy controls

Using MetaForest to further explore sources of heterogeneity, 11 moderators were carried forward at the preselection stage (replicated variable importance metrics shown in Figure S26) for model tuning. The main analysis used 11 candidate variables per split and a minimum of one case per terminal node, which produced positive estimates of explained variance in new data ($R_{oob}^2 = 0.18$; $R_{cv}^2 = 0.66$). The final model identified the following moderators as the most important in explaining the effect size: study year, NOS score, study region, age group, percentage of sample using psychotropic medication, fasting status, mean age, BMI, and illness duration of the AN sample, and mean age and BMI of the HC sample. The relative variable importance of these moderators is shown in Figure S27. The marginal relationship of each moderator with the effect size, averaging over all values of other moderators, is illustrated in Figure S28. These moderators were entered into meta-regressions (Table S2). Only study country was significant, with studies conducted in North America showing a greater difference between AN and HC compared to studies in Europe (B = 0.78; SE = 0.29; z = 2.72; p = 0.007; 95% CI 0.22, 1.34).

Figure S26. Replicated MetaForest for variable preselection, meta-analysis of interleukin-6 (IL-6) concentrations between anorexia nervosa and healthy controls after removing study outliers.

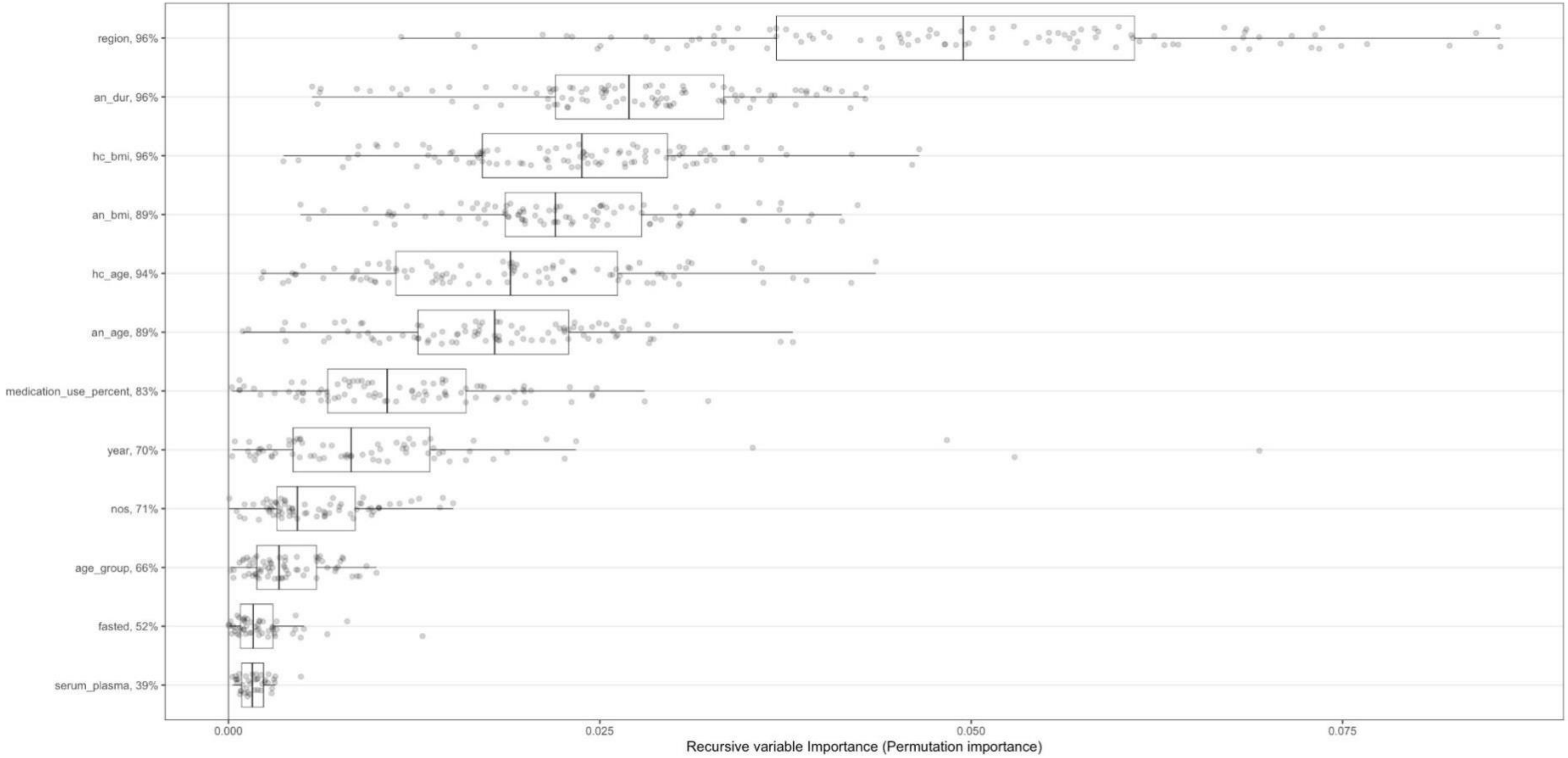

Abbreviations: AN = anorexia nervosa; BMI = body mass index; dur = illness duration; HC = healthy controls; nos = Newcastle-Ottowa Score. Error bars reflect 95% confidence intervals.

Figure S27. Variable importance for final model, meta-analysis of interleukin-6 (IL-6) concentrations between anorexia nervosa and healthy controls after removing study outliers.

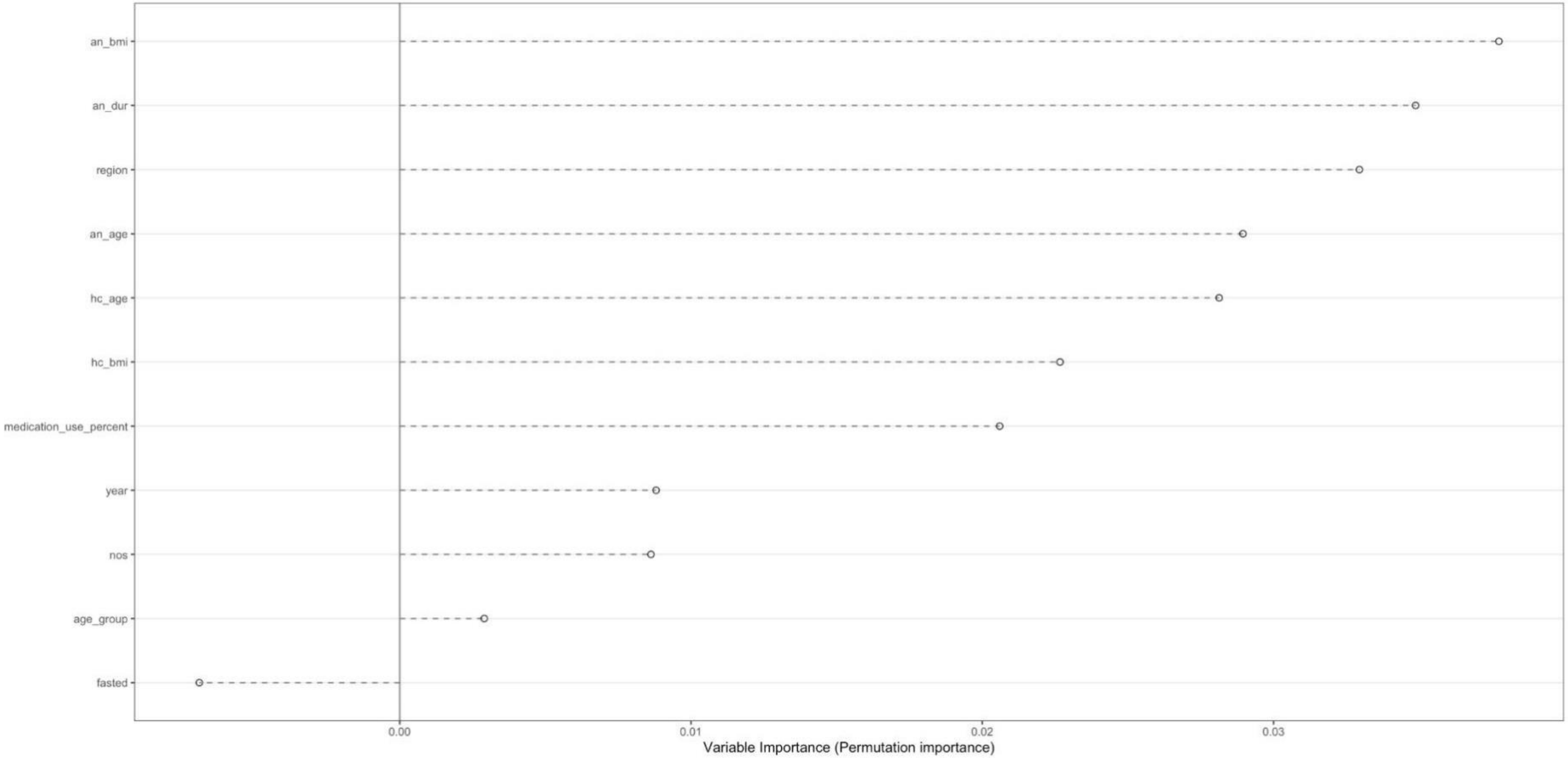

Abbreviations: AN = anorexia nervosa; BMI = body mass index; dur = illness duration; HC = healthy controls; nos = Newcastle-Ottowa Score.

Figure S28. Partial dependence plots, meta-analysis of interleukin-6 (IL-6) concentrations between anorexia nervosa and healthy controls after removing study outliers.

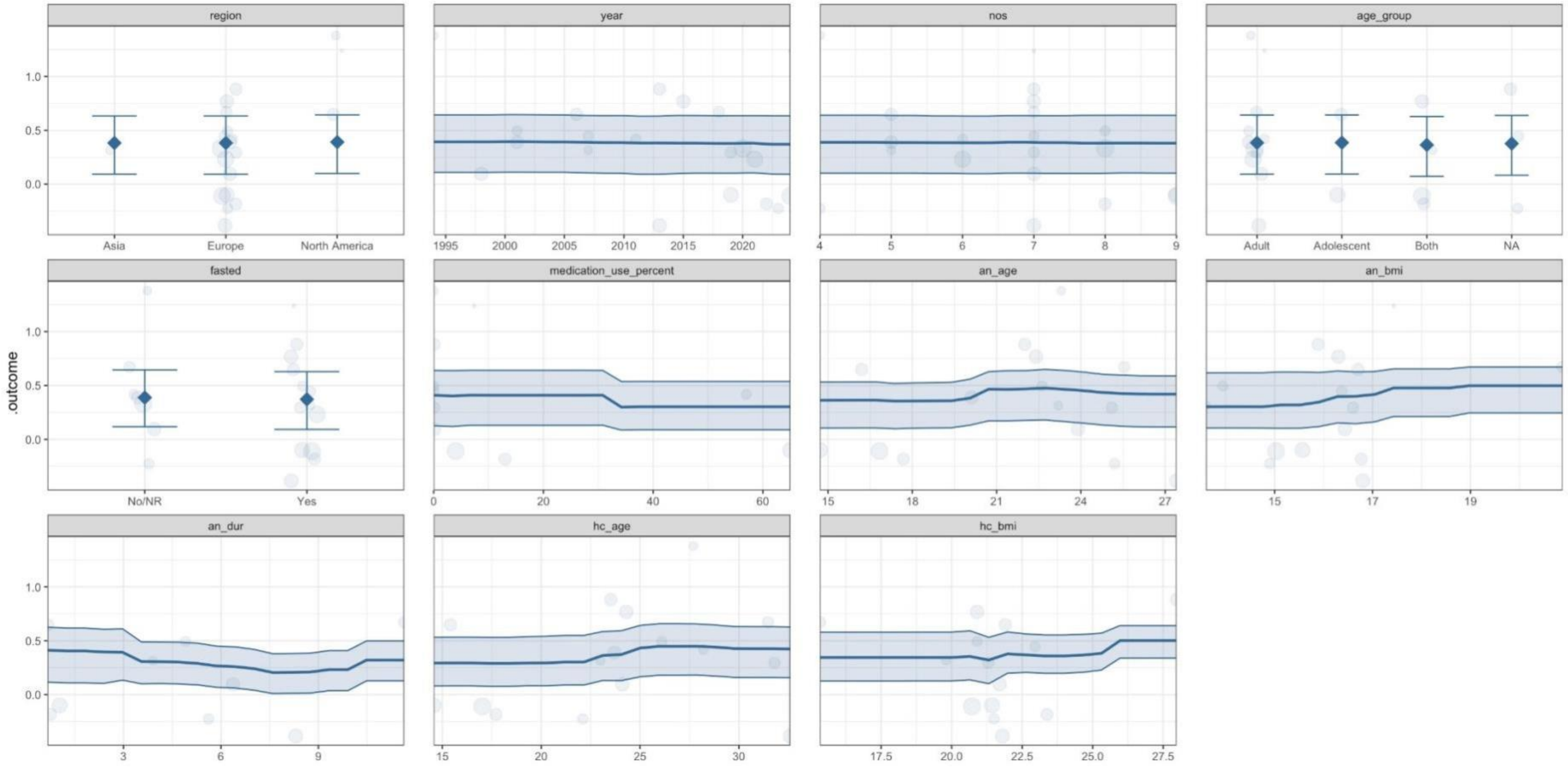

Abbreviations: AN = anorexia nervosa; BMI = body mass index; dur = illness duration; HC = healthy controls; NA = missing; nos = Newcastle-Ottowa Score. Error bars reflect 95% confidence intervals for the model's predictions as each value of the predictor variable.

Table S2. Results of exploratory individual meta-regressions for moderators selected from MetaForest analysis on standardised mean differences in interleukin-6 (IL-6) concentrations between anorexia nervosa and healthy controls, after removing five study outliers [4,5,7,8,10].

Variable	Estimate	SE	z-value	p-value	(95% CI)
Study year	-0.02	0.01	-1.78	0.074	-0.04, 0.002
Study NOS score	-0.11	0.06	-1.77	0.077	-0.23, 0.01
Study region*Asia[a]	0.10	0.50	0.19	0.848	-0.88, 1.08
Study region*North America[a]	0.78	0.29	2.72	0.007**	0.22, 1.34
Percentage of sample who smoke	-0.03	0.03	-0.99	0.324	-0.08, 0.03
Age group*Adults[b]	0.15	0.32	0.47	0.636	-0.48, 0.79
Age group*Both[b]	-0.05	0.36	-0.15	0.884	-0.77, 0.66
Percentage of sample using psychotropic medication	-0.01	0.01	-1.28	0.202	-0.02, 0.003
Fasted	-0.12	0.20	-0.60	0.552	-0.52, 0.28
Mean age (AN)	0.01	0.03	0.39	0.695	-0.05, 0.08
Mean BMI (AN)	0.08	0.08	1.01	0.311	-0.07, 0.23
Mean illness duration (AN)	0.01	0.04	0.23	0.821	-0.07, 0.09
Mean age (HC)	0.02	0.02	0.83	0.406	-0.02, 0.06
Mean BMI (HC)	0.02	0.05	0.24	0.809	-0.08, 0.11

[a]Reference category was Europe. [b]Reference category was adolescents. Abbreviations: AN = anorexia nervosa; BMI = body mass index; CI = confidence intervals; HC = healthy controls; NOS = Newcastle-Ottawa Scale; SE = standard error.

MetaForest moderator analysis for meta-analysis of IL-1β concentrations between anorexia nervosa and healthy controls

To investigate the remaining sources of heterogeneity, a random effects MetaForest analysis was again utilised, using identical parameters. One moderator was carried forward for model tuning (sample type [Plasma/Serum]; replicated variable importance metrics shown in Figure S29). The final model had negative estimates of explained variance in new data (R_{oob}^2 = -0.35). Therefore, there was no evidence for associations between the entered moderators and the SMD for IL-1β.

Figure S29. Replicated MetaForest for variable preselection, meta-analysis of interleukin-1β (IL-1β) concentrations between anorexia nervosa and healthy controls after removing study outliers.

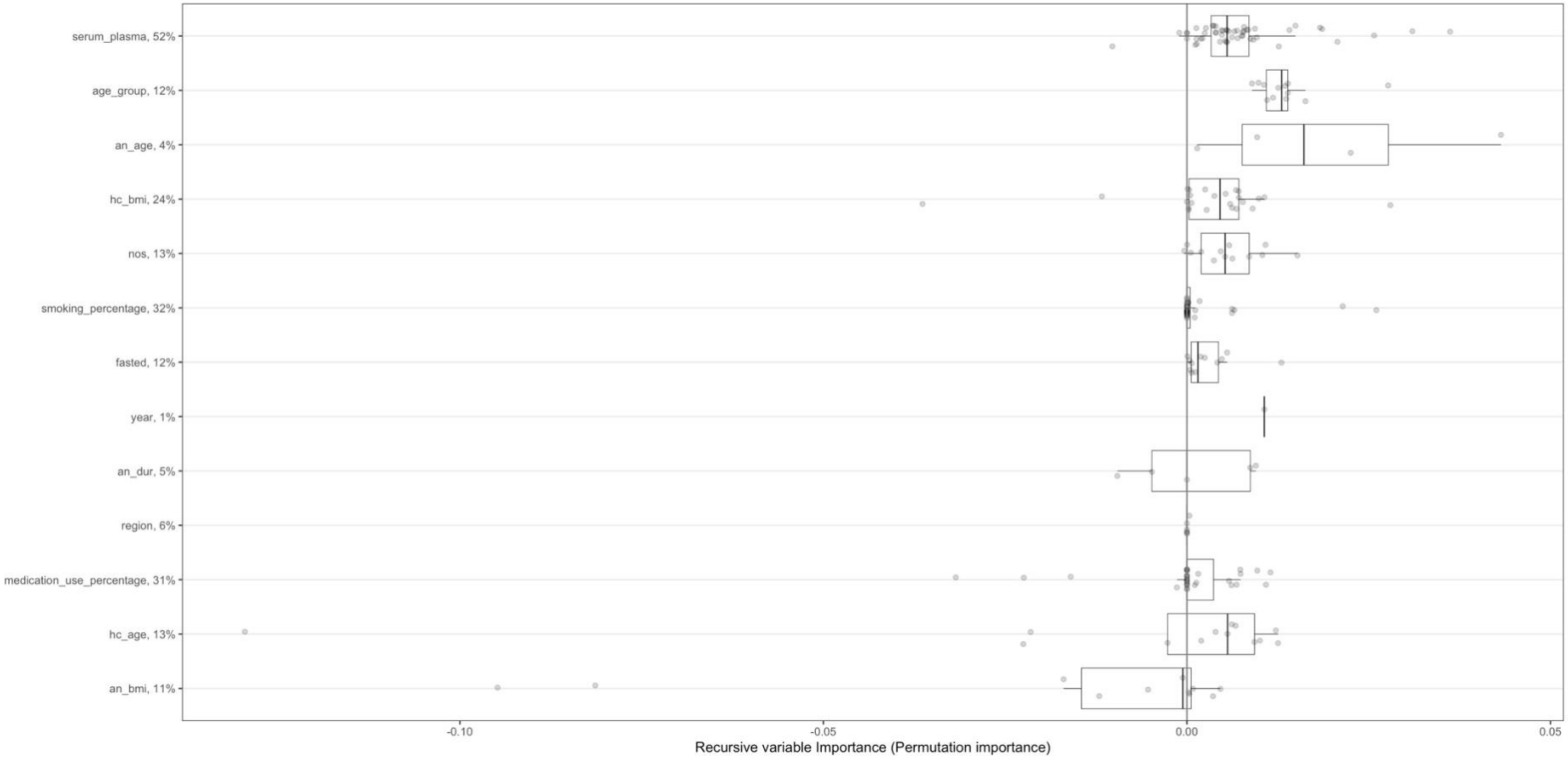

Abbreviations: AN = anorexia nervosa; BMI = body mass index; dur = illness duration; HC = healthy controls; nos = Newcastle-Ottowa Score. Error bars reflect 95% confidence intervals.

Figure S30. Forest plot of standardized mean difference in interleukin (IL)-4, -7, -8, -10 and -15 between AN participants and HCs. Zero is the line of no effect, and points to the right of zero indicate an elevation of the cytokine in AN compared to HCs.

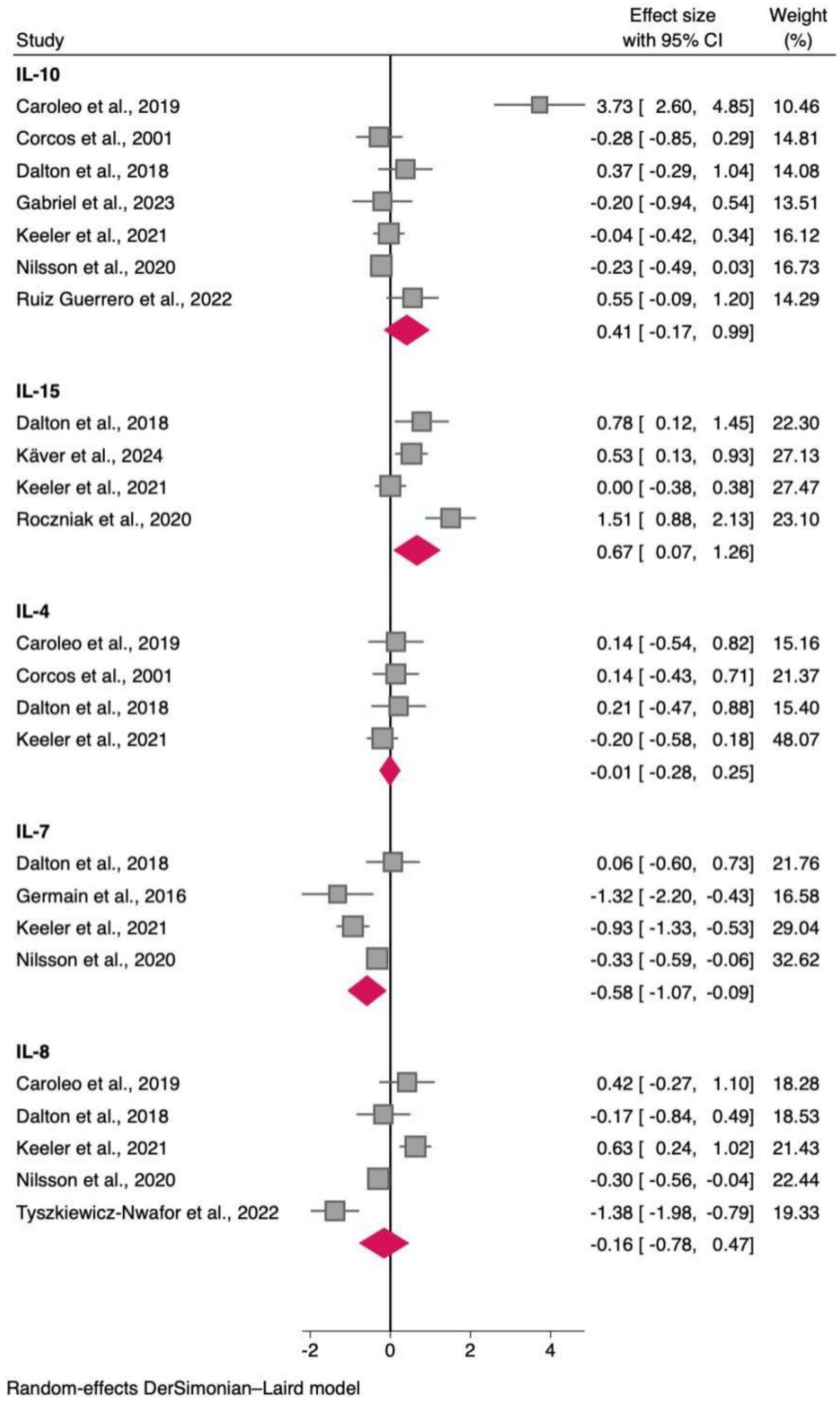

Abbreviations: CI = confidence intervals; IL = interleukin; SMD = standardized mean difference. Error bars reflect 95% CIs.

Figure S31. Forest plot of standardized mean difference in interferon-gamma (IFN-⍰), monocyte chemoattractant protein-1 (MCP) and transforming growth factor-beta (TGF-⍰) between AN participants and HCs. Zero is the line of no effect, and points to the right of zero indicate an elevation of the cytokine in AN compared to HCs.

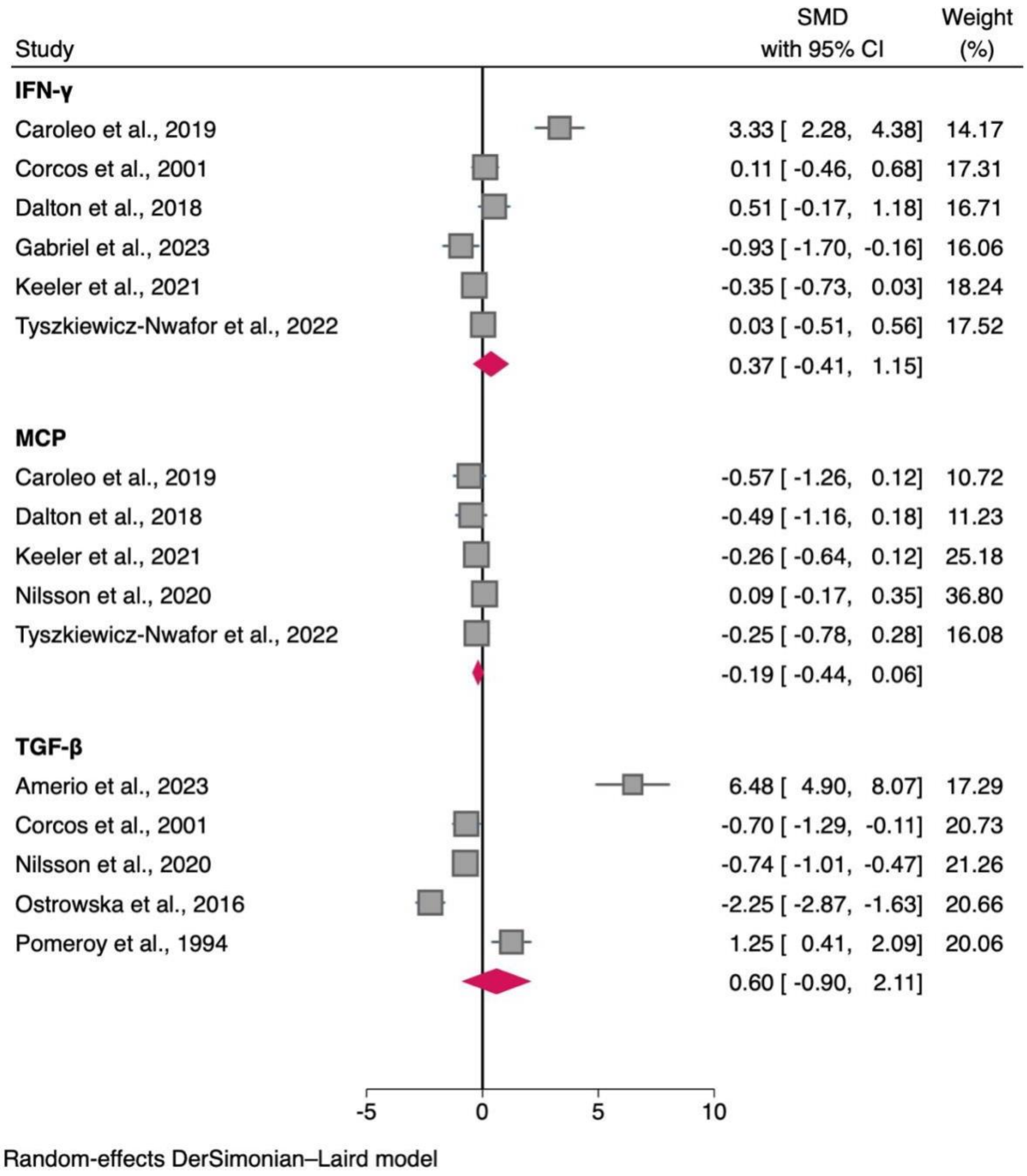

Abbreviations: CI = confidence intervals; SMD = standardized mean difference. Error bars reflect 95% CIs.

Figure S32. Trim-fill funnel plot for cross-sectional meta-analysis of monocyte chemoattractant protein-1 (MCP-1) concentrations between anorexia nervosa and healthy controls.

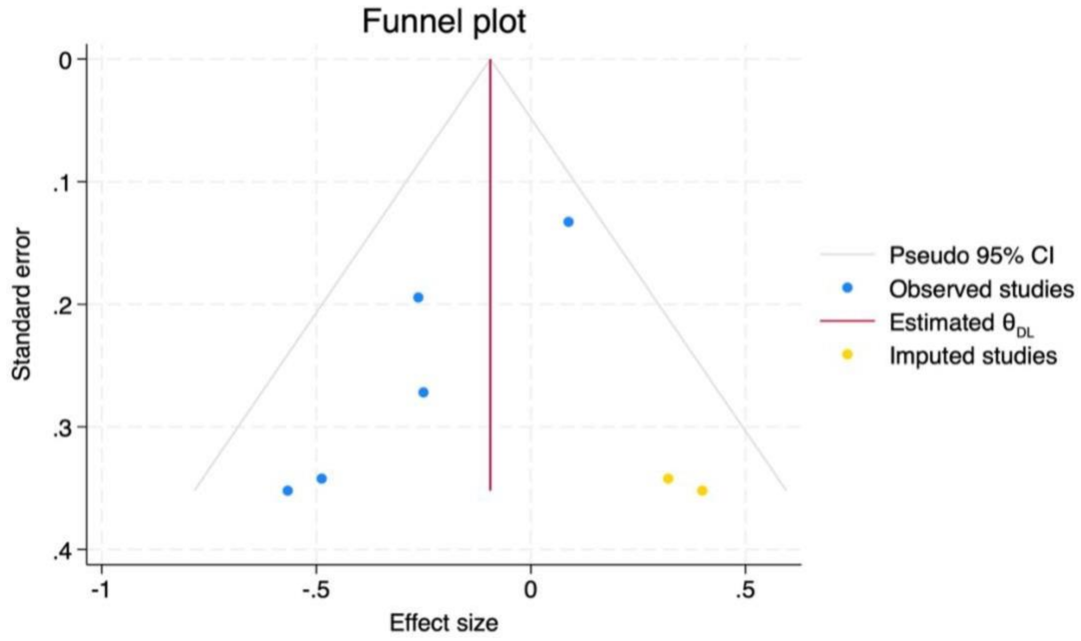

Abbreviations: CI = confidence intervals.

Figure S33. Trim-fill funnel plot for cross-sectional meta-analysis of interleukin-4 (IL-4) concentrations between anorexia nervosa and healthy controls.

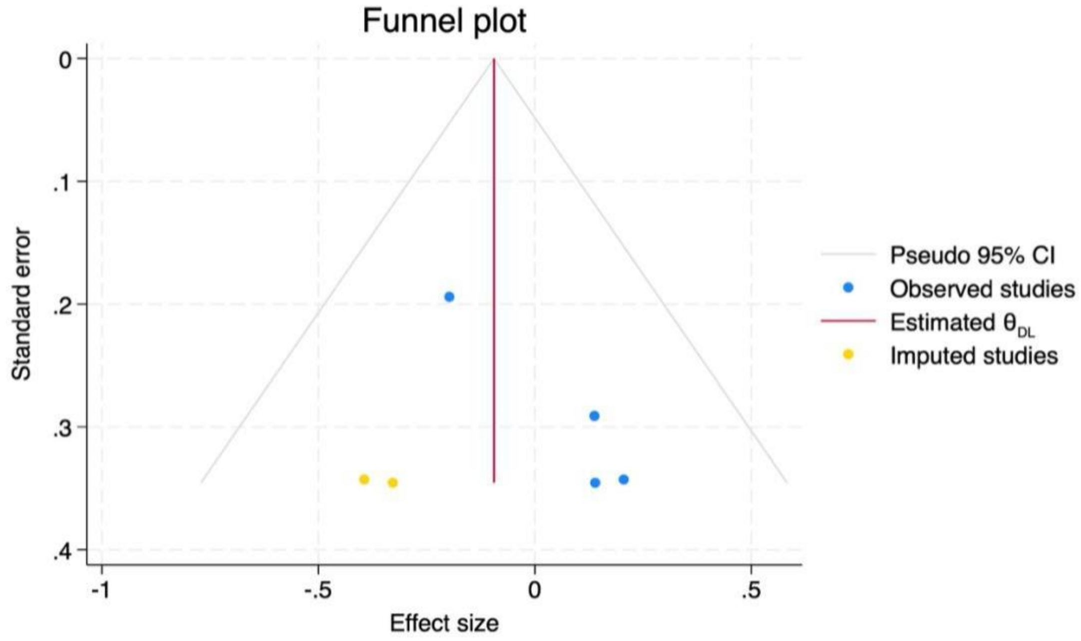

Abbreviations: CI = confidence intervals.

Figure S34. Forest plot of standardized mean difference in tumour necrosis factor-alpha (TNF-α) between AN participants at baseline and follow-up. Zero is the line of no effect, and points to the right of zero indicate an elevation of the cytokine at baseline compared to follow-up.

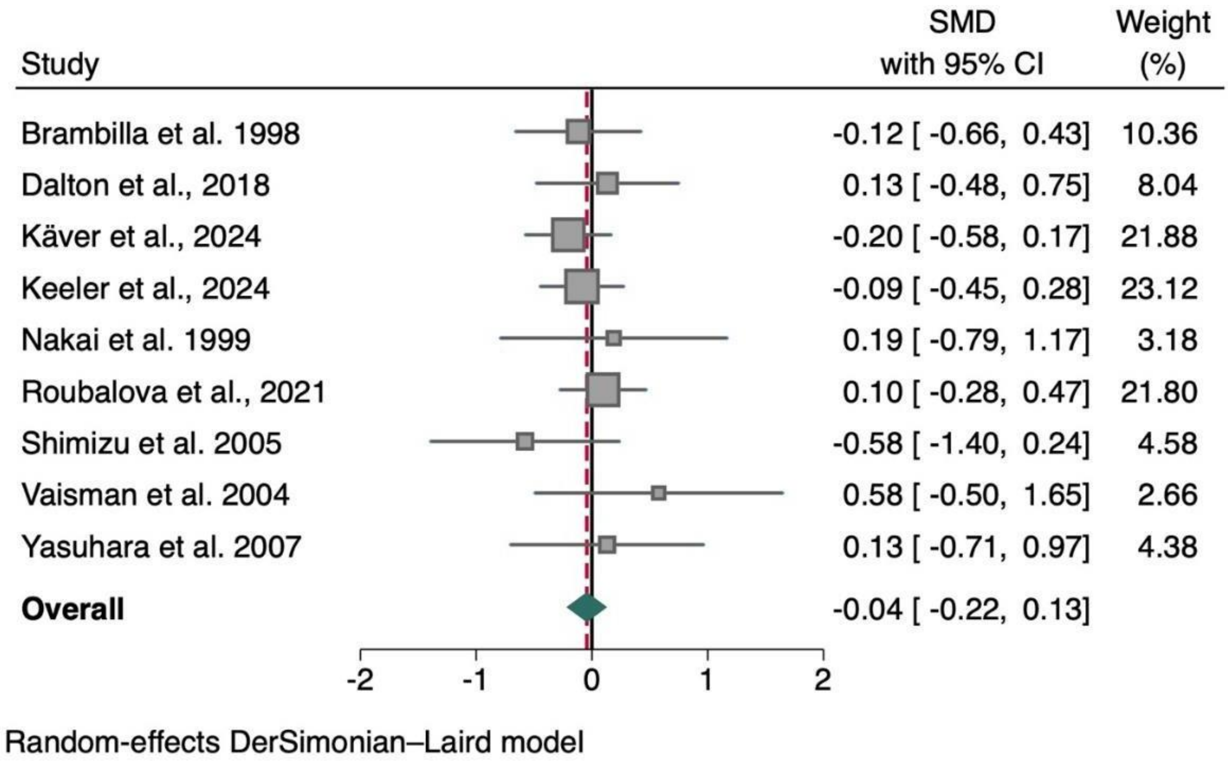

Abbreviations: CI = confidence intervals; SMD = standardized mean difference. Error bars reflect 95% CIs.

Figure S35. Forest plot of standardized mean difference in interleukin-6 (IL-6) between AN participants at baseline and follow-up. Zero is the line of no effect, and points to the right of zero indicate an elevation of the cytokine at baseline compared to follow-up.

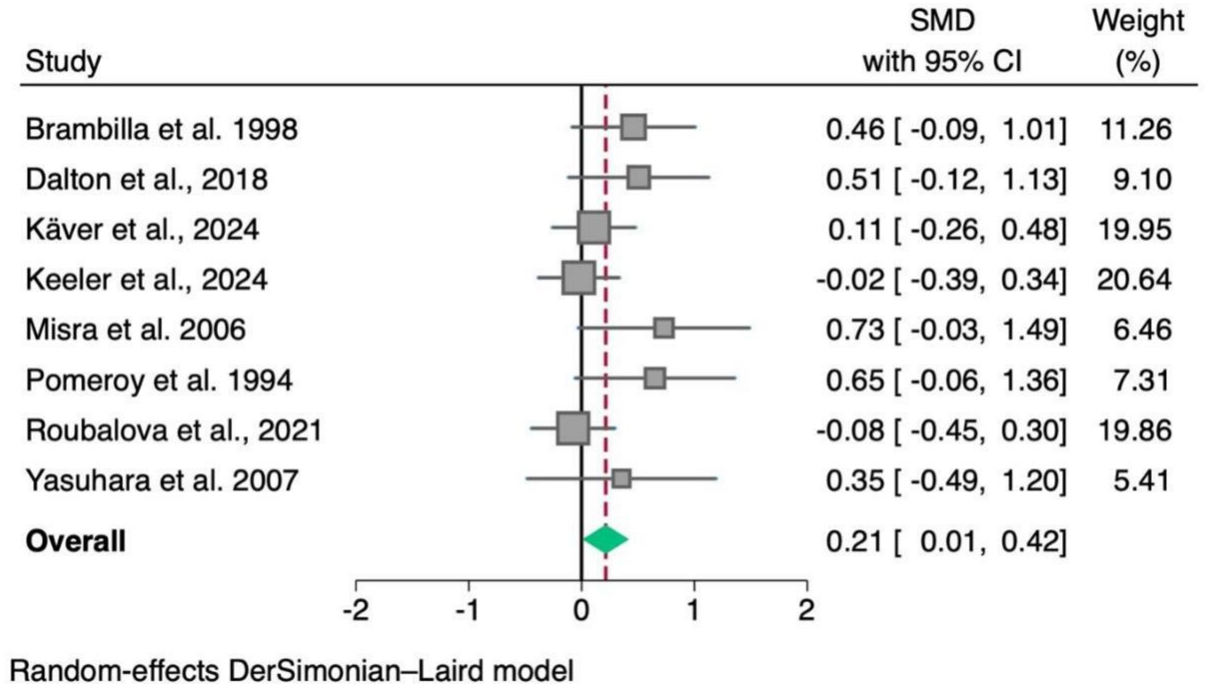

Abbreviations: CI = confidence intervals; SMD = standardized mean difference. Error bars reflect 95% CIs.

Figure S36. Forest plot of standardized mean difference in interleukin-1β (IL-1β) between AN participants at baseline and follow-up. Zero is the line of no effect, and points to the right of zero indicate an elevation of the cytokine at baseline compared to follow-up.

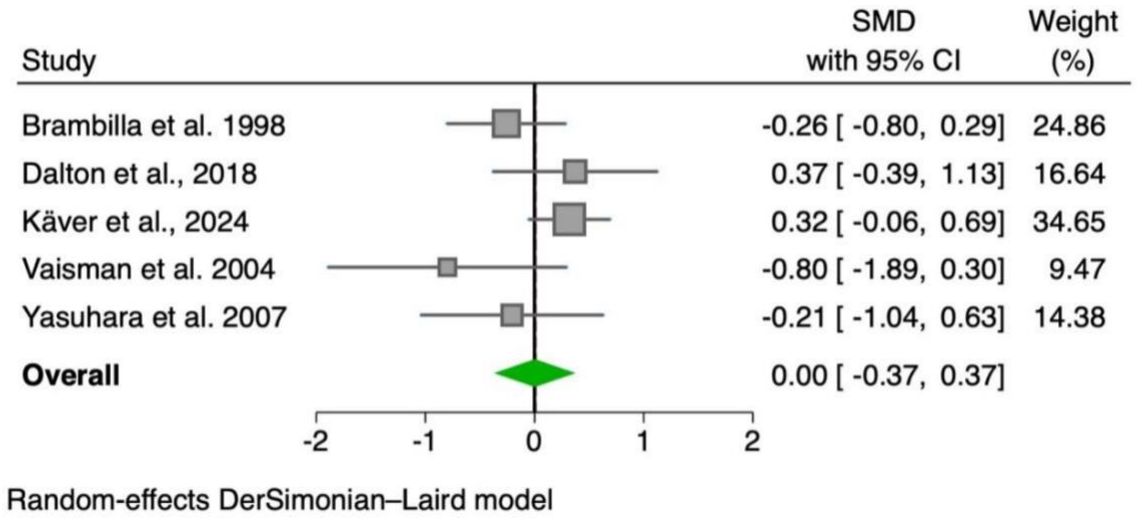

Abbreviations: CI = confidence intervals; SMD = standardized mean difference. Error bars reflect 95% CIs

Figure S37. Trim-fill funnel plot for meta-analysis of tumour necrosis factor-α (TNF-α) concentrations between anorexia nervosa at baseline and follow-up.

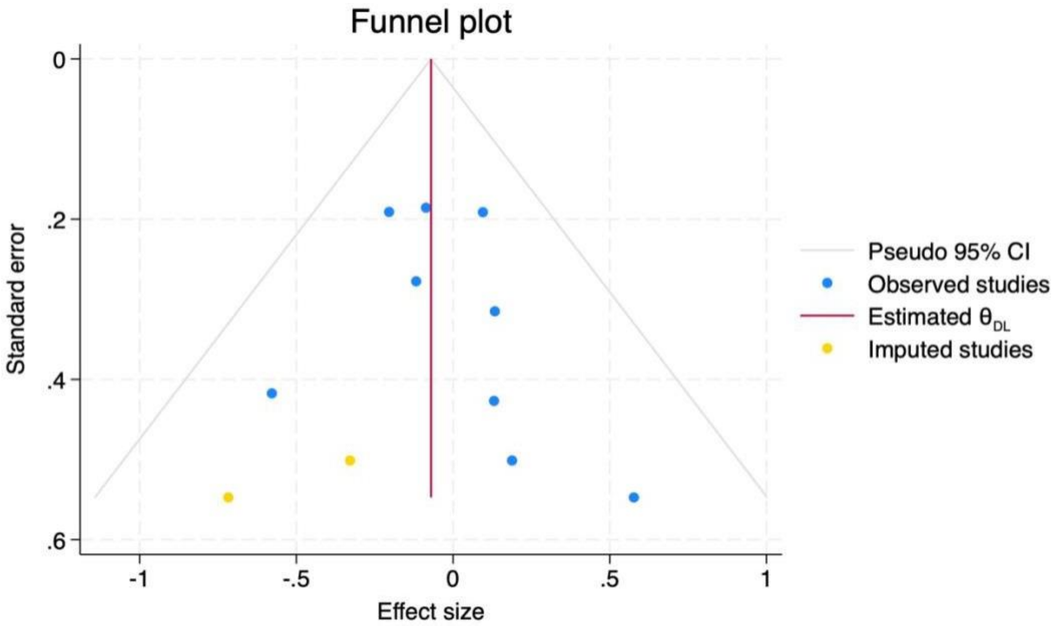

Abbreviations: CI = confidence intervals.

Figure S38. Trim-fill funnel plot for meta-analysis of interleukin-6 (IL-6) concentrations between anorexia nervosa at baseline and follow-up.

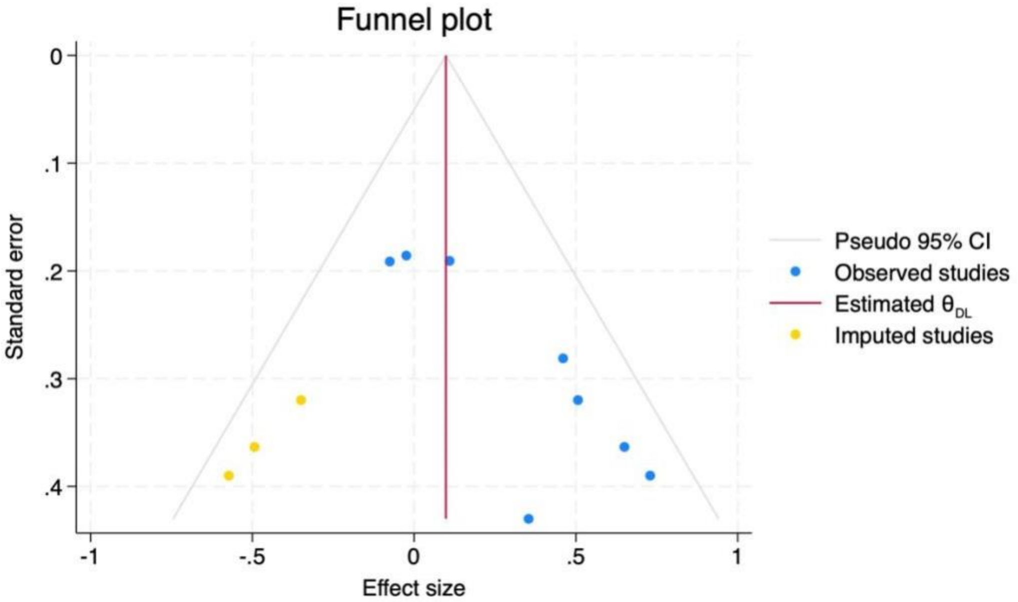

Abbreviations: CI = confidence intervals.

Supplementary references

1 Dalton, B. *et al.* A longitudinal analysis of cytokines in anorexia nervosa. *Brain Behav Immun* **85**, 88-95 (2020). https://doi.org/10.1016/j.bbi.2019.05.012

2 Solis, B. *et al.* The effect of fermented milk on interferon production in malnourished children and in anorexia nervosa patients undergoing nutritional care. *Eur. J. Clin. Nutr.* **56**, S27-S33 (2002).

3 Campanile, A. A., Eckel, L. A. & Keel, P. K. Elevated interleukin-6 in women with binge-eating spectrum disorders. *Int J Eat Disord* **57**, 1510-1517 (2024). https://doi.org/10.1002/eat.24183

4 Amerio, A. *et al.* Dysfunction of Inflammatory Pathways and Their Relationship with Anti-Hypothalamic Autoantibodies in Patients with Anorexia Nervosa. *Nutrients* **15** (2023). https://doi.org/10.3390/nu15092199

5 Keeler, J. L. *et al.* Reduced MIP-1β as a Trait Marker and Reduced IL-7 and IL-12 as State Markers of Anorexia Nervosa. *J Pers Med* **11** (2021). https://doi.org/10.3390/jpm11080814

6 Nakai, Y., Hamagaki, S., Takagi, R., Taniguchi, A. & Kurimoto, F. Plasma concentrations of tumor necrosis factor-α (TNF-α) and soluble TNF receptors in patients with anorexia nervosa. *The Journal of Clinical Endocrinology & Metabolism* **84**, 1226-1228 (1999).

7 Ostrowska, Z. *et al.* Selected pro-inflammatory cytokines, bone metabolism, osteoprotegerin, and receptor activator of nuclear factor-kB ligand in girls with anorexia nervosa. *Endokrynol. Pol.* **66**, 313-321 (2015).

8 Tyszkiewicz-Nwafor, M. *et al.* Expression of immune-related proteins and their association with neuropeptides in adolescent patients with anorexia nervosa. *Neuropeptides* **91**, 102214 (2022). https://doi.org/10.1016/j.npep.2021.102214

9 Víctor, V. M. *et al.* Involvement of leucocyte/endothelial cell interactions in anorexia nervosa. *European journal of clinical investigation* **45**, 670-678 (2015).

10 Käver, L. *et al.* Cytokine and Microbiome Changes in Adolescents with Anorexia Nervosa at Admission, Discharge, and One-Year Follow-Up. *Nutrients* **16** (2024). https://doi.org/10.3390/nu16111596
