# Supplementary material for: Cytokine concentrations in people with eating disorders: A comprehensive updated systematic review and meta-analysis
Source: Commun Med (Lond). 2025 Oct 1;5:408. doi: 10.1038/s43856-025-01122-z (PMC12488998; doi:10.1038/s43856-025-01122-z)
Supplement: Supplementary file 3 — Description of Additional Supplementary files [file 43856_2025_1122_MOESM3_ESM.pdf]

Description of Additional Supplementary files

File name: Supplementary Data 1

Description: Quality assessment of studies included in the systematic review and meta-analysis using the Newcastle-Ottowa Scale adapted for cross-sectional studies

File name: Supplementary Data 2

Description: Study and sample characteristics for studies included in meta-analyses.

File name: Supplementary Data 3

Description: Controlled variables across studies included in the meta-analysis and how they were controlled for (i.e., in eligibility criteria for sample, group matching, analysis, study

File name: Supplementary Data 4

Description: Results of additional studies not included in meta-analysis

File name: Supplementary Data 5

Description: Cytokine data used for meta-analyses
