# Supplementary material for: Cytokine concentrations in people with eating disorders: A comprehensive updated systematic review and meta-analysis
Source: Commun Med (Lond). 2025 Oct 1;5:408. doi: 10.1038/s43856-025-01122-z (PMC12488998; doi:10.1038/s43856-025-01122-z)
Supplement: Supplementary file 9 — Reporting Summary [file 43856_2025_1122_MOESM9_ESM.pdf]

nature portfolio

Corresponding author(s): Johanna Keeler
Last updated by author(s): 21/08/2025

Reporting Summary

Nature Portfolio wishes to improve the reproducibility of the work that we publish. This form provides structure for consistency and transparency in reporting. For further information on Nature Portfolio policies, see our Editorial Policies and the Editorial Policy Checklist.

Statistics

For all statistical analyses, confirm that the following items are present in the figure legend, table legend, main text, or Methods section.

n/a	Confirmed	
☐	☒	The exact sample size (*n*) for each experimental group/condition, given as a discrete number and unit of measurement
☐	☒	A statement on whether measurements were taken from distinct samples or whether the same sample was measured repeatedly
☐	☒	The statistical test(s) used AND whether they are one- or two-sided<br>*Only common tests should be described solely by name; describe more complex techniques in the Methods section.*
☐	☒	A description of all covariates tested
☐	☒	A description of any assumptions or corrections, such as tests of normality and adjustment for multiple comparisons
☐	☒	A full description of the statistical parameters including central tendency (e.g. means) or other basic estimates (e.g. regression coefficient) AND variation (e.g. standard deviation) or associated estimates of uncertainty (e.g. confidence intervals)
☐	☒	For null hypothesis testing, the test statistic (e.g. F, t, r) with confidence intervals, effect sizes, degrees of freedom and P value noted<br>*Give P values as exact values whenever suitable.*
☒	☐	For Bayesian analysis, information on the choice of priors and Markov chain Monte Carlo settings
☒	☐	For hierarchical and complex designs, identification of the appropriate level for tests and full reporting of outcomes
☐	☒	Estimates of effect sizes (e.g. Cohen's *d*, Pearson's *r*), indicating how they were calculated

Our web collection on statistics for biologists contains articles on many of the points above.

Software and code

Policy information about availability of computer code

Data collection	No software was used.
Data analysis	Custom code used based on published code, implemented in R version 4.4.2.

For manuscripts utilizing custom algorithms or software that are central to the research but not yet described in published literature, software must be made available to editors and reviewers. We strongly encourage code deposition in a community repository (e.g. GitHub). See the Nature Portfolio guidelines for submitting code & software for further information.

Data

Policy information about availability of data

All manuscripts must include a data availability statement. This statement should provide the following information, where applicable:

- Accession codes, unique identifiers, or web links for publicly available datasets
- A description of any restrictions on data availability
- For clinical datasets or third party data, please ensure that the statement adheres to our policy

The source data for Figures 2, 3 and 4 are in Supplementary Data 1 (sheets 1, 2 and 3, respectively). The source data for Figure 5 is in Supplementary Data 1, sheet 5. The source data for Figures S30 and S31 is in Supplementary Data 1, sheet 4. The source data for Figures S34, S35 and S36 is in Supplementary Data (sheets 6, 7 and 8, respectively). All other data are available from the corresponding author on reasonable request.

Research involving human participants, their data, or biological material

Policy information about studies with human participants or human data. See also policy information about sex, gender (identity/presentation), and sexual orientation and race, ethnicity and racism.

Reporting on sex and gender	Sex of samples from included studies is noted in the summary table. As data per sex/gender group were not separately reported, it was not possible to report results according to sex/gender. All studies only reported data in females which is stated in the manuscript.
Reporting on race, ethnicity, or other socially relevant groupings	Race and ethnicity are not reported. Genetic ancestry information was unavailable in all studies.
Population characteristics	Age, BMI, current diagnosis, illness duration and treatment were reported.
Recruitment	Studies were selected according to the pre-registered database search, study screening and study selection methods (described in the manuscript and protocol). The recruitment of participants in the individual included studies is assessed for quality against the Newcastle Ottowa Scale criteria.
Ethics oversight	No ethical approval required for a meta-analysis.

Note that full information on the approval of the study protocol must also be provided in the manuscript.

Field-specific reporting

Please select the one below that is the best fit for your research. If you are not sure, read the appropriate sections before making your selection.

☒ Life sciences ☐ Behavioural & social sciences ☐ Ecological, evolutionary & environmental sciences

For a reference copy of the document with all sections, see nature.com/documents/nr-reporting-summary-flat.pdf

Life sciences study design

All studies must disclose on these points even when the disclosure is negative.

Sample size	Meta-analyses can be performed where there are at least two studies available. However, the confidence in the results of such meta-analyses is low, therefore a decision was made that where four or more studies were available for a given cytokine, it would be meta-analysed. Moderator analyses were performed only where there were 10 or more studies available for a given moderator, as per Cochrane guidance.
Data exclusions	Exclusion criteria were pre-established to exclude studies from the meta-analysis. Outliers were also identified in individual meta-analyses and pooled effect sizes were recalculated after their exclusion. Effect sizes were also recalculated after removing studies where >10% of the sample was using psychotropic medication.
Replication	This study was a replication and update of a previous meta-analysis, having broadly similar findings. Differences in the findings are attributable to the inclusion of new studies.
Randomization	Not relevant - meta-analysis.
Blinding	Individual reviewers were blinded to each others' ratings during the screening and quality assessment process.

Reporting for specific materials, systems and methods

We require information from authors about some types of materials, experimental systems and methods used in many studies. Here, indicate whether each material, system or method listed is relevant to your study. If you are not sure if a list item applies to your research, read the appropriate section before selecting a response.

Materials & experimental systems

n/a	Involved in the study	
☒	☐	Antibodies
☒	☐	Eukaryotic cell lines
☒	☐	Palaeontology and archaeology
☒	☐	Animals and other organisms
☒	☐	Clinical data
☒	☐	Dual use research of concern
☒	☐	Plants

Methods

n/a	Involved in the study	
☒	☐	ChIP-seq
☒	☐	Flow cytometry
☒	☐	MRI-based neuroimaging

Plants

Seed stocks	N/A
Novel plant genotypes	N/A
Authentication	N/A
